# A framework for real-time monitoring, analysis and adaptive sampling of viral amplicon nanopore sequencing

**DOI:** 10.3389/fgene.2023.1138582

**Published:** 2023-03-27

**Authors:** Rory Munro, Nadine Holmes, Christopher Moore, Matthew Carlile, Alexander Payne, John R. Tyson, Thomas Williams, Christopher Alder, Luke B. Snell, Gaia Nebbia, Roberto Santos, Matt Loose

**Affiliations:** ^1^ School of Life Sciences, University of Nottingham, Nottingham, United Kingdom; ^2^ DeepSeq, University of Nottingham, Nottingham, United Kingdom; ^3^ BCCDC Public Health Laboratory, Vancouver, BC, Canada; ^4^ Department of Infection, Guy’s and St Thomas’ NHS Foundation Trust, London, United Kingdom; ^5^ Microsoft Research, São Paulo, Brazil

**Keywords:** bioinformactics, software, pipeline, viral sequence analysis, genomics, nanopore sequencing, oxford nanopore minION, oxford nanopore technologies (ONT)

## Abstract

The ongoing SARS-CoV-2 pandemic demonstrates the utility of real-time sequence analysis in monitoring and surveillance of pathogens. However, cost-effective sequencing requires that samples be PCR amplified and multiplexed *via* barcoding onto a single flow cell, resulting in challenges with maximising and balancing coverage for each sample. To address this, we developed a real-time analysis pipeline to maximise flow cell performance and optimise sequencing time and costs for any amplicon based sequencing. We extended our nanopore analysis platform MinoTour to incorporate ARTIC network bioinformatics analysis pipelines. MinoTour predicts which samples will reach sufficient coverage for downstream analysis and runs the ARTIC networks Medaka pipeline once sufficient coverage has been reached. We show that stopping a viral sequencing run earlier, at the point that sufficient data has become available, has no negative effect on subsequent down-stream analysis. A separate tool, SwordFish, is used to automate adaptive sampling on Nanopore sequencers during the sequencing run. This enables normalisation of coverage both within (amplicons) and between samples (barcodes) on barcoded sequencing runs. We show that this process enriches under-represented samples and amplicons in a library as well as reducing the time taken to obtain complete genomes without affecting the consensus sequence.

## 1 Introduction

Oxford Nanopore Technologies (ONT) sequencers (MinION, GridION, Promethion) have allowed sequencing to become a dynamic, real-time process (Jain et al., 2016). By writing batches of sequenced reads to disk after DNA has finished translocating a pore, these data become available immediately, enabling parallel data analysis and so reducing the time required to provide insight into the sequenced sample. Even prior to the ongoing SARS-CoV-2 pandemic, the benefits of real-time analysis of sequence data have been demonstrated (Quick and Loman, 2016; Gardy and Loman, 2018), and rapid lineage assignment and Variant of Concern/Variant under Investigation (VoC/VuI) status can be time sensitive when tracking a new variant (O'Toole et al., 2021).

The ARTIC Network (Quick et al., 2017; Tyson, 2020) (https://artic.network) provides comprehensive protocols for both wet lab and downstream best practice informatics analyses for SARS-CoV-2, amidst other pathogenic viruses. The use of PCR amplification can lead to unequal coverage of individual amplicons in a sequencing library such that some reach sufficient coverage for reliable analysis faster than others. Further sequencing of these amplicons with sufficient coverage will not benefit the final down-stream analysis. Even using 96 barcodes to multiplex samples, the average ONT MinION/PromethION flowcell is capable of providing more data than required. Ideally, sequencing would be stopped as soon as sufficient data are available for analysis with balanced coverage of amplicons in the library. Aside from wet lab optimisations, ONT sequencers offer Run Until, the ability to stop sequencing once some pre-defined condition has been met, and adaptive sampling (Payne 2020), the ability to stop sequencing and unblock off target DNA from the pore, which may help address these problems.

As part of the COG-UK network (Cog, 2020; Nicholls et al., 2021) we generated thousands of SARS-CoV-2 consensus sequences using ONT sequencers. To test the utility of run until in this context, we incorporated the ARTIC pipeline into our minoTour tool (Munro et al., 2021) (https://github.com/looselab/minotourapp) and developed a model to predict if sufficient coverage will be obtained for each barcoded sample on a flowcell, stopping sequencing when all samples predicted to achieve sufficient coverage do so. We demonstrate this has no effect on the ability to assign lineages (O'Toole et al., 2021) to samples and minimal impact on SNP calls. The resultant shorter sequencing runs preserve flow cell health, allowing them to be flushed and reused for other experiments, reducing the effective cost per sample for sequencing.

**FIGURE 1:**
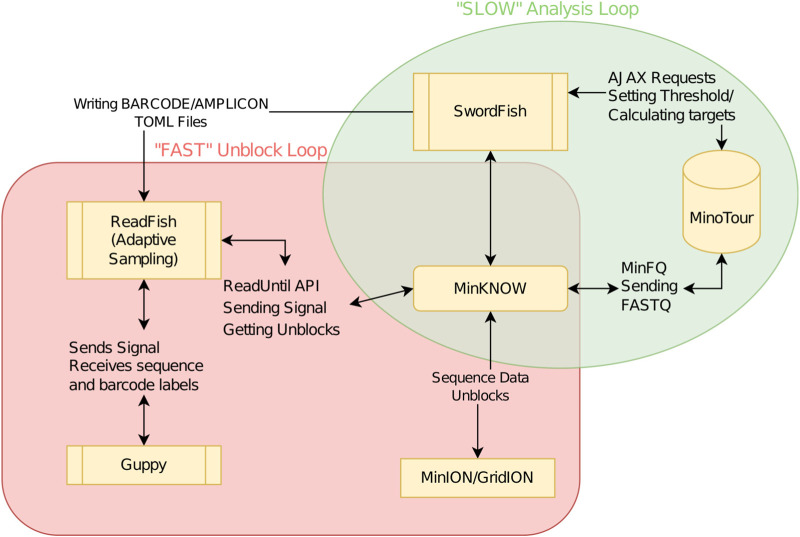
Flowchart demonstrating ReadFish/SwordFish/minoTour interactions. The slow analysis loop (green) is used to update ReadFish’s target TOML file. The loop is run once every 60 s. MinFQ uploads FASTQ sequence data to minoTour, which tracks coverage for each amplicon on each barcode. SwordFish queries minoTour for the set of amplicon coordinates on each barcode to unblock. These are defined as those exceeding a specified level of coverage (e.g., 50×). SwordFish updates a TOML file that can be read by ReadFish. The fast analysis loop (red) is run every read batch (approximately 0.8 s). ReadFish updates its coordinates from the TOML file, base calls and demultiplexes all reads in the batch using Guppy, and then sends unblock signals to MinKNOW for any reads that align inside any amplicon with sufficient coverage.

To determine if adaptive sampling could be used to select individual amplicons from one or more samples to improve and balance the coverage across SARS-CoV-2 genomes we developed SwordFish. This tool enables truly “dynamic” adaptive sampling by providing feedback between minoTour and the ReadFish pipeline (https://github.com/looselab/swordfish). SwordFish couples ReadFish to minoTour by querying minoTour for information on specific sequencing runs and updating ReadFish (Payne et al., 2021) with new barcode/amplicon targets in response to ongoing data generation (see Figure 1). Using a custom 1,200 base pair amplicon scheme (Supplementary File S3.5) we show adaptive sampling can filter out over abundant samples and individual amplicons, and coupled with run until, results in time savings and an increase in the number of amplicons reaching median 20× coverage.

## 2 Implementation

### 2.1 MinoTour ARTIC pipeline implementation

The standard ARTIC pipeline uses Nanopolish (Loman et al., 2015; Quick et al., 2017) for signal level analysis of raw ONT data during variant calling. Unfortunately, signal level data are unavailable within minoTour. Instead we integrated ARTIC’s alternate Medaka (https://github.com/nanoporetech/medaka) workflow to enable real-time generation of consensus genomes during sequencing. MinoTour’s workflow contrasts with other web based analysis platforms which either do not exploit the real-time features of the nanopore platform or do not have access to the sequence data themselves for further analysis (Bruno et al., 2021; Ferguson et al., 2021). The ARTIC network does provide a tool, RAMPART, which can monitor a run over time and complete analysis for individual samples, but does not provide many of the other features shown here at this time, such as lineage analysis or adaptive sampling. (https://artic.network/rampart). We integrated the ARTIC SARS-CoV-2 Medaka pipeline into minoTour as a custom python script, which is run as a Celery task (https://docs.celeryproject.org/en/stable/), processing read batches as they are uploaded. The pipeline is asynchronous, preventing blocking of any other analyses being performed. Uploaded reads are filtered by length, with the minimum and maximum read lengths permissible calculated from the underlying amplicon scheme. Reads are further filtered by the QC score assigned by Guppy (assigned pass by Guppy) and then mapped to an appropriate SARS-CoV-2 reference using minimap2 (Li, 2018). Per base coverage is tracked in optimised numpy arrays using the mapped reads in real-time (Walt and Varoquaux, 2011).

Coverage is tracked for each individual amplicon on a sample as defined by the primer scheme in use. Default parameters for triggering the analysis of a specific sample are at least 90% of the amplicons (completeness) covered at a median depth of at least 20×, though these are user configurable. Once triggered, the accumulated mapped reads for that sample are passed to the ARTIC network’s Medaka pipeline. Numerous primer schemes can be chosen, including custom schemes, simply by creating the appropriate primer scheme and reference files and uploading them to minoTour.

MinoTour uses pangolin (O'Toole et al., 2021) to assign a PANGO lineage from the most recent lineage classifications. Consensus sequences are also compared with current VoC/VuI definitions as defined at https://github.com/phe-genomics/variant_definitions, using the Aln2Type tool (https://github.com/connor-lab/aln2type). Both PANGO lineages and VoC/VuI designations are automatically updated daily by minoTour. A report is generated for each sample (see Supplementary Figures S1A, B) and optionally users can be notified of VoC/VuI identifications *via* the minoTour Twitter API. Sequences within each run are also globally aligned using MAFFT (Katoh et al., 2002), with an illustrative tree generated using iQ-Tree (Minh et al., 2020) and visualised with figtree.js or ToyTree (Rambaut, 2021). Additional background sequences can be included in these trees if desired and the distribution of SNPs within consensus sequences from the run, compared with the reference are displayed in a SNIPIT plot (https://github.com/aineniamh/snipit) (Supplementary Figure S1A).

Results from the pipeline are maintained for historical record, with files stored on disk and metadata and metrics about the ARTIC sequencing experiment stored in a SQL database. These results are then visualised in the minoTour web server. Once a run has completed, which is automatically recognised by the fact that no further data are added to the flow cell within a fixed period of time, all analyses are automatically re-run to ensure maximum coverage for consensus generation. A retention policy for sequence data is set globally for the site and all read data can be automatically scrubbed from the server after consensus generation, if desired.

### 2.2 ARTIC visualisations and reports

If running an ARTIC analysis on a flow cell, minoTour provides a custom page containing all ARTIC data and visualisations (Supplementary Figures S1A–D). This page shows the performance of all samples in the run and then visualises detailed performance and information available for an individual sample. A sortable and searchable summary table shows users metrics about each sample in the run, with average coverage, number of amplicons at different depths and basic statistics such as mean read length and read count. If the sample had sufficient data to be run through the ARTIC pipeline, we display the assigned lineage and VoC/VoI status.

Further details can be seen for a chosen sample such as per base coverage plots for the sample genome. Assigned PANGO lineage information is provided in tabular form, with links out to further information describing each lineage (https://cov.lineages.org and https://outbreak.info). The VoC/VuI report generated by Aln2Type is visualised and the final status assigned displayed. A PDF report for each barcoded sample and the overall run can be exported, showing all above metrics for each sample. An example can be found in Supplementary File S3.1.

Pass and fail VCF files, BAM files and pangolin lineages can be downloaded. Optionally, these features can be disabled and minoTour will remove all files that may contain identifiable sequence information from the server. By maintaining compatibility with standard ARTIC bioinformatics pipelines, this tool can be adapted to run any ARTIC compatible pathogen analysis simply by uploading the appropriate reference files.

### 2.3 Amplicon coverage prediction model

To predict if individual samples are likely to result in an informative genome sequence, providing the basis for minoTour’s decision on when to stop the run, minoTour assumes the user is seeking minimal useful genome completeness (default 90% amplicons with at least 20× median “pass” read coverage). Using median coverage depth reduces the impact of small insertions/deletions on monitoring amplicon coverage. In addition, median coverage is only calculated for unique regions of each amplicon, removing any overlap between amplicons. This prevents amplicons with more than 50% overlap being incorrectly labelled as complete due to the coverage of a neighbouring amplicon. MinoTour then assumes that each ONT flow cell can generate a minimum of 100,000 reads for each sample detected and so projects whether each sample will reach minimal useful completeness using a simple model (Equation 1). A sample is projected to finish if 90% of the amplicons have a predicted final coverage over the minimum required coverage (default 20×). All sequencing runs gather data for 1 h before any of our strategies are used to ensure reasonable sampling of the loaded library.

$$\left(\frac{\text{Amplicon median coverage}}{\text{Total mapped reads}}\times \text{Barcodes identified}\times 100,000\right)\geq \text{Min. required coverage}$$ (1)

### 2.4 SwordFish–real-time readfish target updating software

Swordfish provides a python based command line interface to connect minoTour to ReadFish *via* minoTour’s Representational State Transfer (REST) Application Programming Interface (API), querying for updates at a user specified interval. In the context of amplicon based sequencing, SwordFish receives a list of barcodes and amplicon genomic coordinates for each barcode from minoTour, where the median coverage for any returned amplicon exceeds the user defined threshold. SwordFish then adds the coordinates of these over coverage amplicons to the rejection targets for the correct barcode in ReadFish’s configuration file. ReadFish will then reject any future reads corresponding to that amplicon. If a barcoded sample has completed analysis, SwordFish can switch off that barcode entirely for the remainder of the experiment. The relationship between minoTour, swordfish and ReadFish is shown more clearly in Figure 1. It is worth noting that whilst this manuscript focuses on SARS-CoV-2, this approach is applicable to any viral amplicon primer scheme that can be used with the ARTIC field bioinformatics pipeline provided the amplicons are sufficiently long and ligation, not rapid, sequencing is used. If rapid kit based sequencing were to be used, the amplicons would have to be of sufficient size to generate a library with a long enough mean read length that the software would have time to unblock them.

### 2.5 Post run genome analysis

**FIGURE 2:**
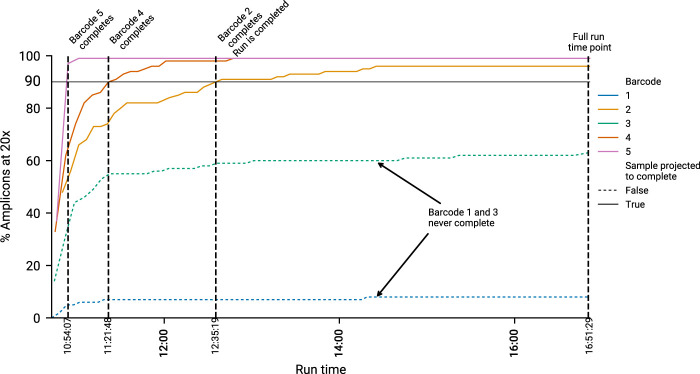
An example run, showing how timepoints are calculated. Five barcodes are displayed. Black horizontal line indicates the 20× coverage on 90% of amplicons threshold. The time at which a barcode reaches this threshold is recorded as the “Sample Completion” (SC) time point. Two illustrative samples, barcode 1 and 3, are not predicted to finish, and do not cross this threshold. Hence they have no associated SC time. Once all barcodes predicted to finish are complete, we record the “Run Completed” (RC) time point. This is the time minoTour would recommend stopping the run. The Full Run time is when the run stopped without any early intervention.

To determine how manipulating run time affects results, we defined three time points of interest for a sample during a sequencing run. The Full Run time point, the Run Complete time point and the Sample Complete time point. Full Run is defined as the time at which the run completed with no intervention. Run Complete is the point in a run where all samples our algorithm predicted would complete (90% completeness, 20×) had done so. Finally, Sample Complete is the point at which an *individual* sample in a run reached sufficient completeness and is automatically put through the ARTIC pipeline by minoTour, whilst the run continues. A sequencing run will have only one Full run time point, one Run complete time point, but will have many Sample Complete time points. This concept is visualised in Figure 2.

To create consensus genomes from time points equivalent to our ARTIC pipeline and compare the results of both Medaka and Nanopolish we had to calculate the sets of both the signal (FAST5) and FASTQ files equivalent to those that would have been uploaded to minoTour at each of the time points. We iteratively mapped all reads from each barcode across 13 reference ARTIC runs using minimap2 (Li, 2018), in FASTQ file creation order, creating cumulative alignment files. Using mosdepth (Pedersen and Aaron, 2018) we determined cumulative coverage at each base across the reference genome, for each FASTQ file creation time point, and then the median coverage for each amplicon using the same primer scheme based approach as in minoTour. This identifies the time points in each run when sufficient data are available to trigger minoTour to analyse the samples, as well as the points that minoTour would have recommended stopping the run based on it is amplicon coverage predictions. The creation time point for the FASTQ file that results in sufficient coverage to meet any appropriate thresholds was used to identify the time in the sequencing run when analysis would occur. Using this method, we can identify the equivalent FAST5 file for that FASTQ file from the ONT sequencing summary file, enabling us to analyse the data with both Medaka and Nanopolish (code available from https://github.com/LooseLab/artic_minotour_analyses). For each time-point, we generated consensus FASTA files to calculate genome recovery, defined as the proportion of non N positions in the final sequence. This is a close approximation of the minoTour completeness metric, as any base that has 20× coverage going into the ARTIC Medaka pipeline will most likely be called as non N.

## 3 Results and discussion

### 3.1 Amplicon coverage prediction model

**FIGURE 3:**
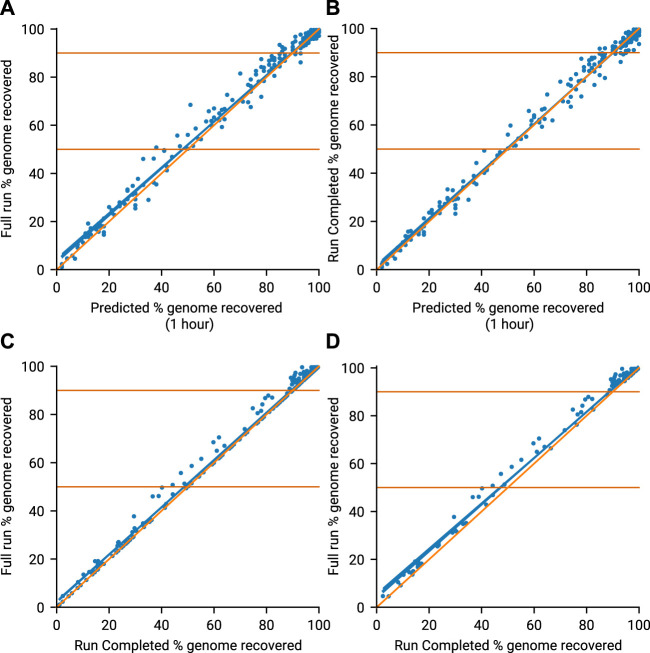
Genome recovery throughout all 13 runs. Blue line (line of best fit), Orange line (*x* = *y*). **(A)** Compares the predicted genome recovery based on 1 hours sequencing (*X*-axis) with the actual coverage seen at the end of the run (*Y*-axis). Predicted recovery is the proportion of amplicons in a sample expected to reach 20× coverage. Actual coverage is the proportion of non N bases in the consensus genomes obtained at the end of the run (Full Run). **(B)** Compares the same predicted genome recovery with the actual coverage observed at the Run Complete time as defined by minoTour. **(C)** Compares the actual coverages reported in A (Full Run) and B (Run Complete). **(D)** Is the same as C but ignores those runs where the Run Complete time is the same as the Full Run time.

The amplicon prediction model performed well across all runs (Figure 3A, *R* ^2^ = 0.991). The model proved to be conservative, slightly under-predicting against final coverage, which prevented minoTour from waiting for genomes to complete which would never do so. After an hour of data, predicted genome recovery collection compares well with that observed at the calculated Run Complete times for the 13 runs (Figure 3B). The strong correlation (*R* ^2^ = 0.993) between predicted values and values actually recovered provides confidence in our algorithm. Comparing the genome recovery achieved at the Run Complete time point with the genome recovery seen at Full Run (Figure 3C) shows some small further benefits in recovery (R^2^ = 0.996) when allowing the run to reach natural completion. This is expected as continuing the run for longer allows the missing 10% of each genome to acquire some further coverage. However the longer a run continues the more it is information return diminishes, so stopping earlier accelerates time to answer as well as allowing the flow cell to be reused and save costs. This can be seen more clearly in Figure 3D, (R^2^ = 0.994) when filtering out those runs where no time is saved by our model, as these runs have the same time defined for Run Complete and Full Run.

Our model confidently predicts if a sample will generate sufficient data to provide useful information with enough accuracy to support a decision on whether or not to continue sequencing. There is a potential small loss in data as a consequence of reducing the sequencing time. We therefore quantify the consequences of this on time saved, lineage assignment and SNP calling below.

### 3.2 Run until time savings

**FIGURE 4:**
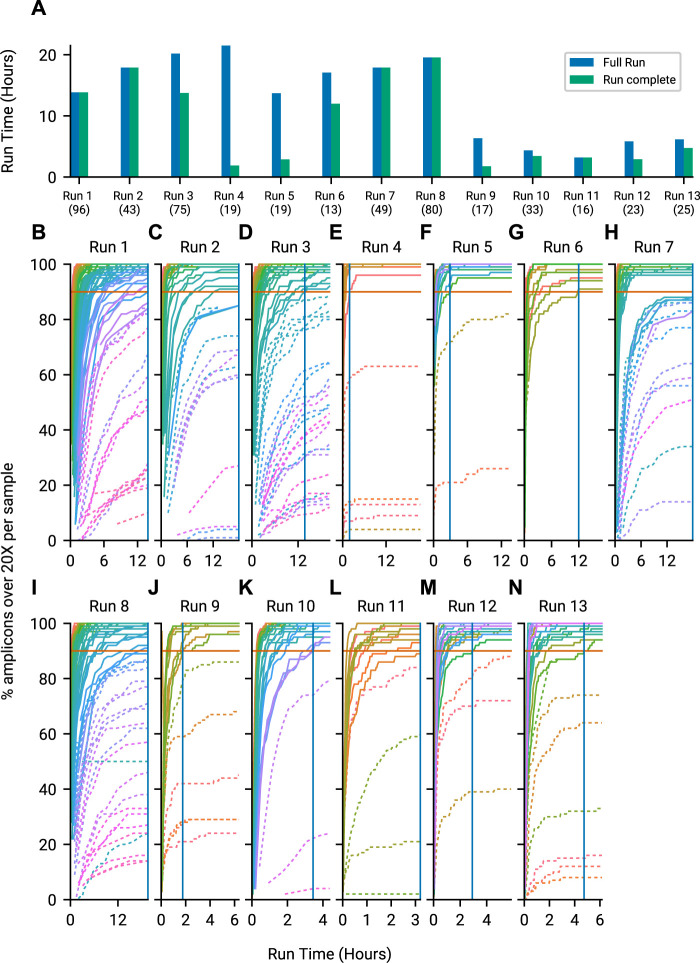
Time savings by using minoTours ARTIC Pipeline and amplicon coverages for each run, across the course of the run. **(A)** The Full Run time point plotted against minoTours Run complete time point as hours since the run started, for each run. Number of samples shown in brackets below the run label. **(B–N)** Samples across 13 runs showing the percentage of amplicons at 20× over time. Barcodes that we project to finish are displayed with solid lines, whilst barcodes we project not to finish are dashed. 90% (Our threshold for firing) is marked on each plot. Once all barcodes that are projected to finish cross the 90% threshold, we would instruct MinKNOW to stop the run. This time is marked by a solid blue vertical line.

To quantify whether this approach results in useful time savings, we tracked metrics and predicted amplicon coverages per barcoded genome sample using minoTour for 13 sequencing runs. We visualised a comparison of calculated Run Complete time and Full Run time in Figure 4A. Runs 9 through 13 were actively monitored with minoTour and manually stopped at earlier run times in response to the model predictions, resulting in the shorter Full Run length and the similarly quick Run Complete time point. Time savings using this approach are dependent on the sample composition, but are often significant (for example, Run 4, Figure 4A). By plotting all barcodes on every run, we can visualise the point in time in a run when all barcodes predicted to finish in a run cross the threshold. Negative controls are treated as a sample, and are predicted to fail, so they do not prevent a run from completing. Time savings are greater in runs with fewer samples as each has relatively more sequencing capacity available as can be seen in Figures 4B–N.

### 3.3 SNPS and lineages

Finally we investigated whether stopping early affected the information you can retrieve from consensus genomes, and compared whether minoTour loses SNP accuracy by using the Medaka pipeline rather than Nanopolish.

#### 3.3.1 Lineage assignment to consensus genomes

Across all 13 sequencing runs, a total of 508 SARS-CoV-2 samples were sequenced (including negative and positive controls). The number of genomes produced by the ARTIC Pipeline at each time point were: Full run, 456 genomes; Run Completed, 454 genomes; Sample Completed, 334 genomes. The two additional genomes produced at the Full Run time over the Run Complete time are both extremely low completeness genomes (only 1% of the genome has consensus sequence) that failed to call at the Run Complete time. Across all time points for any given sample in any run, we observe complete concordance in lineage assignment between either Medaka or Nanopolish generated genomes (Supplementary File S3.2). Any loss of data seen by stopping sequencing early did not impact PANGO lineage assignment in a SARS-CoV-2 sequencing run. We note that these sequences are predominantly from the B.1.1.7 lineage due to the time periods in which they were collected, but given our observations on SNP calling below do not envisage this being an issue.

#### 3.3.2 Comparing SNPs between Medaka and Nanopolish consensus genomes

We compared Nanopolish and Medaka consensus genome sequences for all genomes in our data set (1,245 genomes across Full Run, Run Complete and Sample Complete time points from 508 unique samples). The SNPs were called using nextclade (https://clades.nextstrain.org) with the output data available in Supplementary Files S3.3, S3.4.

**FIGURE 5:**
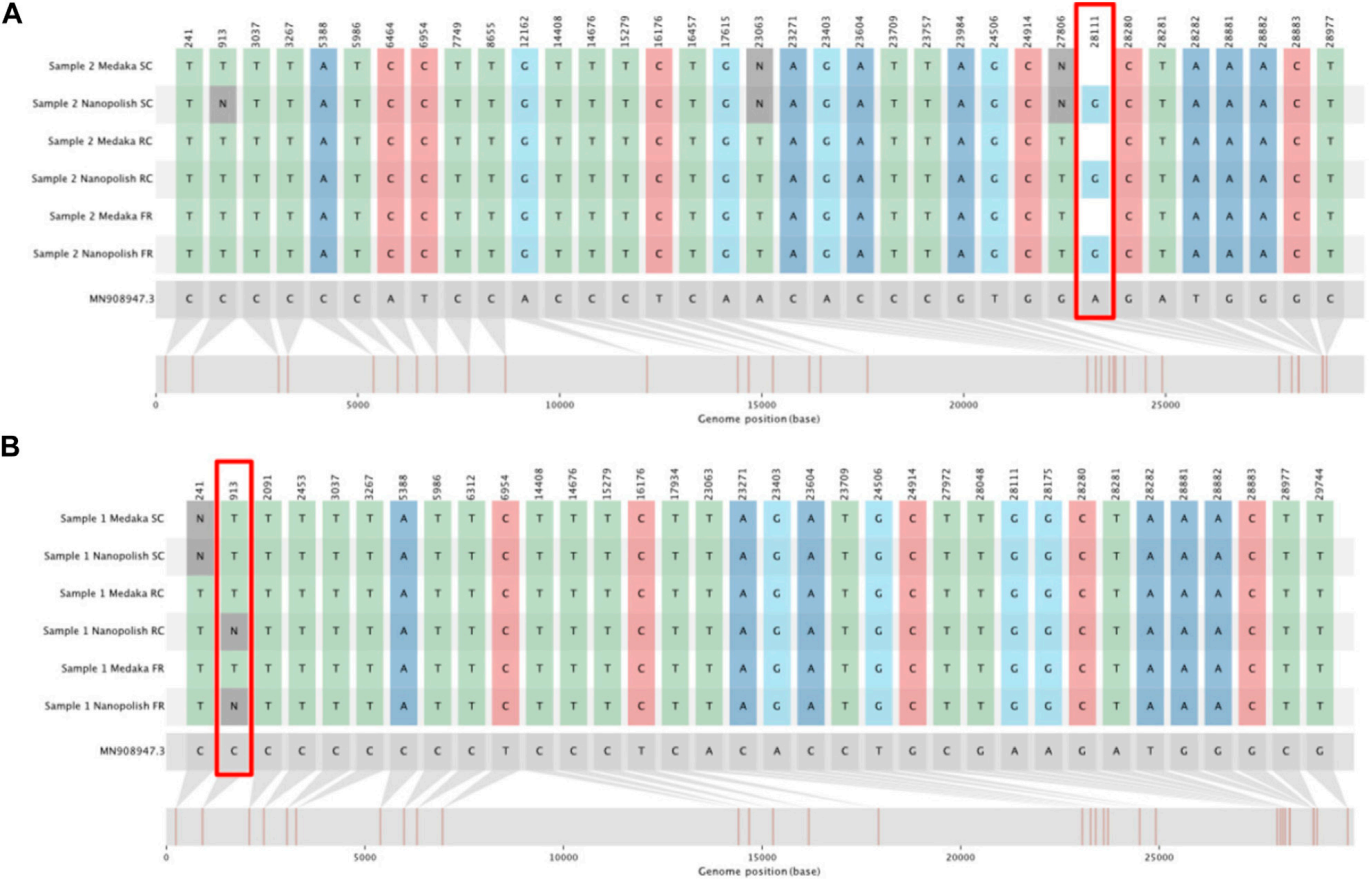
SNIPIT plots demonstrating particularly divergent positions for SNP calls between Medaka and Nanopolish. The tracks from top to bottom show SNPs as called from consensus genomes for the Sample Complete time, Run Until and Full run time points (in this order) for both the Medaka and Nanopolish pipelines. **(A)** SNIPIT plot showing an example pair of consensus genomes with Nanopolish calling a SNP at position 28111 but Medaka calling reference. **(B)** SNIPIT plot, showing the Nanopolish pipeline switch from a SNP to an N at position 913 with more data, on a single sample across our three time points.

Of the 456 genomes generated at the Full Run time-point, 341 called SNPs identically whether they were generated by Medaka or Nanopolish. The majority of the remaining genomes either Medaka or Nanopolish are unable to confidently call a site and so assigns an ambiguous base (N), altering the SNP call. Of more concern, there are some sites which are incorrectly assigned as a reference call by Medaka (Table 1), 27 total. Upon inspection, the majority of these are for one single site in the genome at position 28,111 (Figure 5A). We also note one site, 913, for which Nanopolish rarely can call a SNP at lower coverage, but changes to an ambiguous call at higher depth, however this is a very unusual case. It is very infrequent that an increase in coverage over 20× alters a call. An example of this is illustrated at Figure 5B.

**TABLE 1:** Contingency table comparing SNP calling between Medaka and Nanopolish for all three time points. Displayed are total counts across all sites called as either reference (Ref), SNP or unknown (N). Only genomes present in each category (Full Run, Run Completed and Sample Completed) are included in the analysis.

	Medaka									
		Full Run			Run completed			Sample completed		
		N	SNP	Ref	N	SNP	Ref	N	SNP	Ref
Nanopolish	N	151,292	29	418	183,709	32	412	837,973	116	253
	SNP	25	10,727	5	26	10,697	6	78	9,984	13
	Ref	24	0	9,818,580	33	0	9,786,167	37	2	9,132,765

At the Run Complete time point, there is an increase in the number of ambiguous (N) sites called by both pipelines, most likely a consequence of the slightly lower coverage data available (Tables 1, 2). However overall the difference between calls made in consensus genomes generated by both pipelines at this time point is very slight (99.7% identical calls). It is worth mentioning that as this is the time point that genomes would finish in a minoTour ARTIC run, we conclude that there is very little effect in using Medaka in our pipeline. There is a very slight increase in the number of SNPs being called by one pipeline being called as an N in the other (1 for Nanopolish SNPs and 3 for Medaka SNPs), although again this is likely due to slightly lower coverage.

**TABLE 2:** Contingency table displaying the mean count for sites called as either reference (Ref), SNP or unknown (N), for all three time points (Full Run, Run Completed and Sample Completed), for each SNP calling pipeline. Note, The total of each column, excluding (N. genomes) represents every position in a SARS-CoV-2 genome. Only genomes present in each category (Full Run, Run Completed and Sample Completed) are included in the analysis.

	Medaka			Nanopolish		
	Full run	Run complete	Sample completed	Full run	Run complete	Sample completed
N	453	550	2,509	454	551	2,510
SNP	32	32	30	32	32	30
Ref	29,398	29,301	27,345	29,397	29,300	27,343
N. Genomes	334	334	334	334	334	334

The Sample Complete time genomes are of lower quality, with approximately a 5 fold increase in the number of Ns seen in a generated consensus genome (Table 2). However we note that only one sample finishes at this time point in an actual run (the last to reach our completion threshold). When comparing Nanopolish and Medaka genomes at the Sample Complete time point, we can see that there is a very small increase over the Run Complete time point generated genomes in disagreement between the SNP calls (0.00013% of all calls). However the calls are effectively concordant even at this earliest time point, and as previously noted, only one generated genome actually finishes at this time point in an actual run.

Finally we compared the genomes that did not reach our completion threshold in our run, thus lacking a Sample Complete time point. As shown in Table 3, these genomes are of much lower quality, and do not improve by allowing the run to continue to the Full Run length. They are approximately 48% Ambiguous N calls on average, and there is no gain in the average number of SNPs called.

**TABLE 3:** Contingency table displaying the mean count for sites called as either reference (Ref), SNP or unknown (N). Note, The total of each column, excluding (N. genomes) represents every position in a SARS-CoV-2 genome. Only genomes NOT present in the Sample completed category are included in the analysis.

	Medaka		Nanopolish	
	Full Run	Run Until	Full Run	Run Until
N	14,519	14,813	14,495	14,813
SNP	19	19	19	19
Ref	15,353	15,059	15,374	15,056
N. Genomes	123	120	123	120

Thus we conclude the majority of SNP call differences between Medaka and Nanopolish are differences in ambiguous calls. Overall, we conclude that Medaka is sufficient for variant calling and lineage assignment, but in our downstream analysis workflows we routinely run both pipelines for confirmation.

### 3.4 SwordFish based adaptive sampling

Sequencing libraries were prepared using the standard ARTIC protocol and a custom set of 1,200 base pair primers (Freed, 2020) (BED file available in Supplementary File S3.5). Sequencing was monitored in real-time using minoTour (Munro et al., 2021) and SARS-CoV-2 samples analysed using minoTours ARTIC pipeline. ReadFish at commit 0ccb5932 (https://github.com/LooseLab/readfish/tree/0ccb59324906635a0d077f94d7f82388039885cb) was used to perform targetted sampling, as unlike ONTs adaptive sampling, experimental configurations can be updated during a run (Payne et al., 2020). MinKNOW was configured to provide data in 0.8 s chunks. Sequencing was performed on a GridION Mk1 (ONT). The method requires Guppy version 4.2 or later for barcode de-multiplexing. Basecalling was performed using the HAC model for final analysis with “require both ends” for de-multiplexing set to true. ReadFish was configured to use fast base calling, requiring barcodes at one end. Starting configuration TOMLs and commands can be found at https://github.com/looselab/swordfish-experiments.

In our first demonstrative experiment, we selected a range of representative clinical samples (see Supplementary Table S1) with Cycle threshold (Ct) values ranging from 14 to 30, as well as some samples for which no Ct values were available. We utilised the standard ARTIC requirement for reads being barcoded at each end and sequenced using the LSK109 library protocol (ONT). Barcoding at both ends undoubtedly favours downstream analysis as rejected reads will only possess a single starting barcode, and so are assigned as unclassified, Even so, this approach provides an ideal test for throughput and the performance of ReadFish. We ran three separate experiments on our flow cell, visualised in Supplementary Figure S2. The library was not normalised prior to loading, and four barcodes were clearly abundant 58, 64, 76, 88, when no adaptive sampling was applied to the library Supplementary Figure S2A. We began by unblocking based purely on barcode assignment Supplementary Figure S2B. Unexpectedly, even given the 1200bp read lengths, this resulted in the ability to detect 16 more amplicons at 50× coverage on the less abundant barcodes, compared with the control run after 110 min of sequencing, as shown in Supplementary Table S2.

We next used SwordFish to update ReadFish’s targets in real-time, based on real-time analysis by minoTour, to provide granular control of individual amplicon/barcode combinations. Using the same library as our previous experiment, we applied a simple threshold approach rejecting reads from amplicon/barcode combinations once coverage exceeded 50×. More sophisticated algorithms for normalisation, for example probabilistic discard, could be considered but would have to account for the large dynamic range of amplicon concentration in samples. As shown in Supplementary Table S2, it is possible to individually address each amplicon/barcode combination to ensure the total coverage does not exceed a predetermined threshold. Inspection of the relative change in amplicon/barcode proportion reveals that some amplicons within abundant barcodes are themselves effectively enriched, suggesting that this targeted approach is better than simple inactivation of entire barcodes. The relative change in proportion of classified amplicon/barcode combinations is slight, as expected for the short amplicons sequenced here (Supplementary Figure S3C, Supplementary Figure S3E and Supplementary Figure S3G). Enrichment efficiency is further reduced by short fragments present within these libraries (Supplementary Figures S4, S5).

**FIGURE 6:**
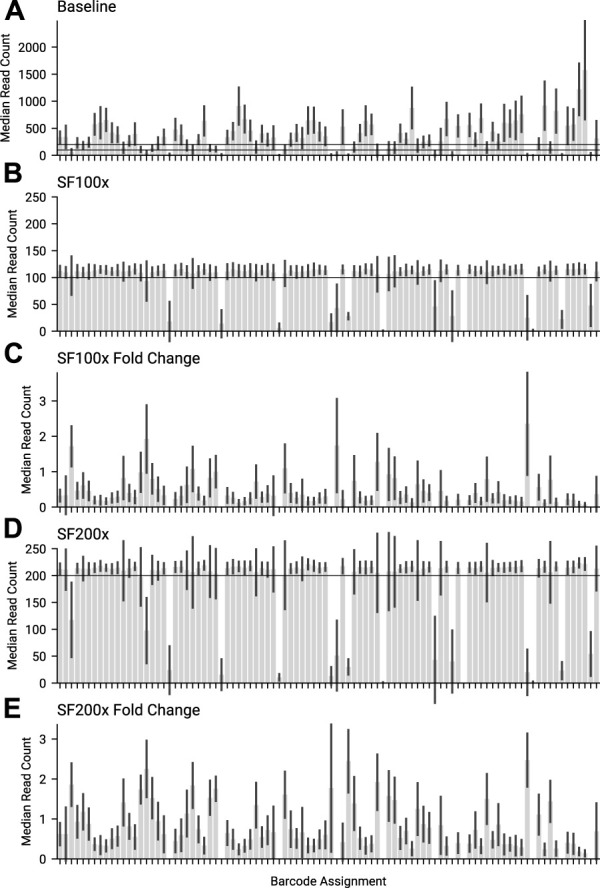
The median amplicon read count for each barcode, for the same library across different swordfish threshold targets. **(A)** Baseline, where no adaptive sampling was applied. 100 and 200 are marked as the targets for the other 2 experiments. **(B)** SF100 had a 100× coverage swordfish threshold target. 100× is marked on the graph. **(C)** Fold change for the median amplicon read count per barcode, between SF100x and Baseline. **(D)** SF200 had a 200× coverage threshold target. 200 is marked on the graph. **(E)** Fold change for the median amplicon read count per barcode, between SF200x and Baseline.

**FIGURE 7:**
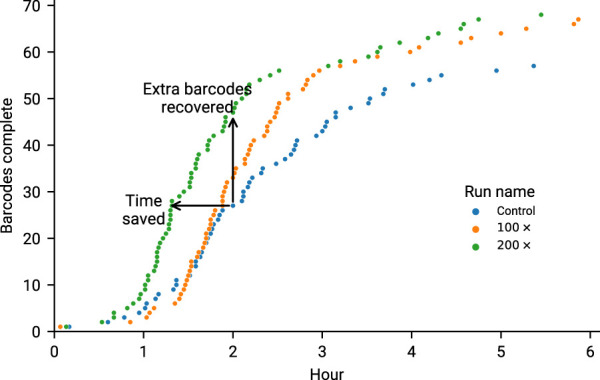
Overlapped runs in our second experiment (6 h of sequencing) marking the time at which a barcode reached 90% of amplicons at 20×. It is worth noting the increased performance of 200× is likely due to the increased yield, as this was the first run to go on. Time saved is considered to be points for a run that are shifted left of their equivalents on the *y*-axis. Any points above the Control run (at the same time) are considered as extra barcodes recovered.

The current maximum number of barcodes in a library available for nanopore sequencers is 96, at the time of writing. We proceeded to test our approach against the maximum number of samples, targeting 200× coverage of each sample, running for 6 h on a MinION Mk1b. Given that our amplicon primer scheme has 29 amplicons, we are tracking a total 2,784 unique amplicon/barcode combinations. We then ran the same library targeting amplicon coverage of 100× for a further 6 h. Finally we ran a 6 h control experiment, with no adaptive sampling. The median amplicon coverages for each sample achieved are displayed in Figure 6. As each condition was run on the same flowcell, after a nuclease flush and reload, there was a small decrease in total sequence yield for each experiment (Supplementary Table S3). Potential maximum enrichment was again brought down by the presence of some short material in the library (Supplementary Figure S6), but ReadFish displayed sufficient performance to keep up with 96 barcodes, with the unblocked read length falling far short of the sequenced. Inspecting some illustrative barcodes (unclassified, 32, 62, 92, 93), we see that indeed our analysis bins all unblocked reads into unclassified, as shown in Supplementary Figure S7. Figure 7 illustrates that using adaptive sampling with ReadFish/Swordfish resulted in an increase in the number of amplicons reaching useful coverages, as well as accelerating the time at which these amplicons reached this coverage. We also see an increase in the number of amplicons that we recover in the SwordFish enabled runs, recovering up to 108 more amplicons at 50× coverage when compared to the control run, as shown in Supplementary Table S4. It is worth noting that these amplicons may have reached this coverage in the control run with more time, as there was a smaller yield in our control run due to having had two experiments run on the flowcell beforehand. Thus, although the effects are relatively small, this approach of individually addressing each amplicon on each barcode in a 96 barcode library will benefit the sequencing run.

In a third set of experiments, Swordfish/Readfish was applied to the midnight protocol 1,200 base pair amplicon scheme using the RBK110.96 rapid library preparation kit, and sequenced on a GridION Mk1. As anticipated, the rapid protocol results in reads shorter than the amplicon length and so we saw no benefit as either a filter to balance barcodes or the speed at which amplicons reached completion Supplementary Figures S8A–C, S9. This experiment was run in triplicate.

Overall, applying adaptive sampling to ARTIC SARS-CoV-2 sequencing reveals the fundamental challenges of enriching short material. Reads must be long enough to benefit from time saved by rejecting unwanted reads. Effectively this application is more of a simple filter to remove unnecessary excess reads with minimal enrichment benefits. Longer read lengths would improve enrichment capabilities, but are less useful for viral amplicon sequencing due to the risk of drop out. In the future, as flow cell yield increases, and these features become available on the PromethION, this approach will enable dynamic adjustment of yields obtained from individual samples in barcoded libraries. The model presented here relies on real-time analysis of the data obtained to determine if an experimental objective has been achieved. Any method that does not consider the final data risks bias as a result of unexpected read length distribution differences between barcoded samples.

## 4 Conclusion

We demonstrate that by reducing the run time for a SARS-CoV-2 sequencing run using real-time analysis to calculate the best stopping point, it is possible to balance flow-cell health and time to answer while minimising any information loss. Significant time savings are possible using this approach; this has previously been described as “Run Until”, a method described by Oxford Nanopore Technologies but to date, not widely used. We show that stopping a run at the earliest point where sufficient data are available does not negatively affect subsequent downstream analysis. In addition, Read Until provides further benefits to a SARS-CoV-2 sequencing run, by reducing the number of unnecessary reads in the analysis, reducing the time taken to complete individual genomes and focusing sequencing capacity on incomplete samples. Real-time analysis in conjunction with adaptive sampling demonstrates powerful balancing of amplicon coverage on up to 96 samples, even providing limited enrichment in some cases. In order for this method to work, amplicons must be sufficiently long. Whilst the current maximum barcode number is 96, we anticipate this approach being able to handle many more samples.

## Data Availability

The original contributions presented in the study are publicly available. Source code and documentation is available at https://github.com/LooseLab/minotourapp and https://github.com/LooseLab/swordfish Supplementary data are available from https://github.com/LooseLab/artic_minotour_analyses.
