# Supplementary material for: A framework for real-time monitoring, analysis and adaptive sampling of viral amplicon nanopore sequencing
Source: Front Genet. 2023 Mar 27;14:1138582. doi: 10.3389/fgene.2023.1138582 (PMC10083257; doi:10.3389/fgene.2023.1138582)
Supplement: Supplementary file 1 [file DataSheet1.ZIP › supplementary_data/Supplementary_Info.pdf]

1 Supplementary figures

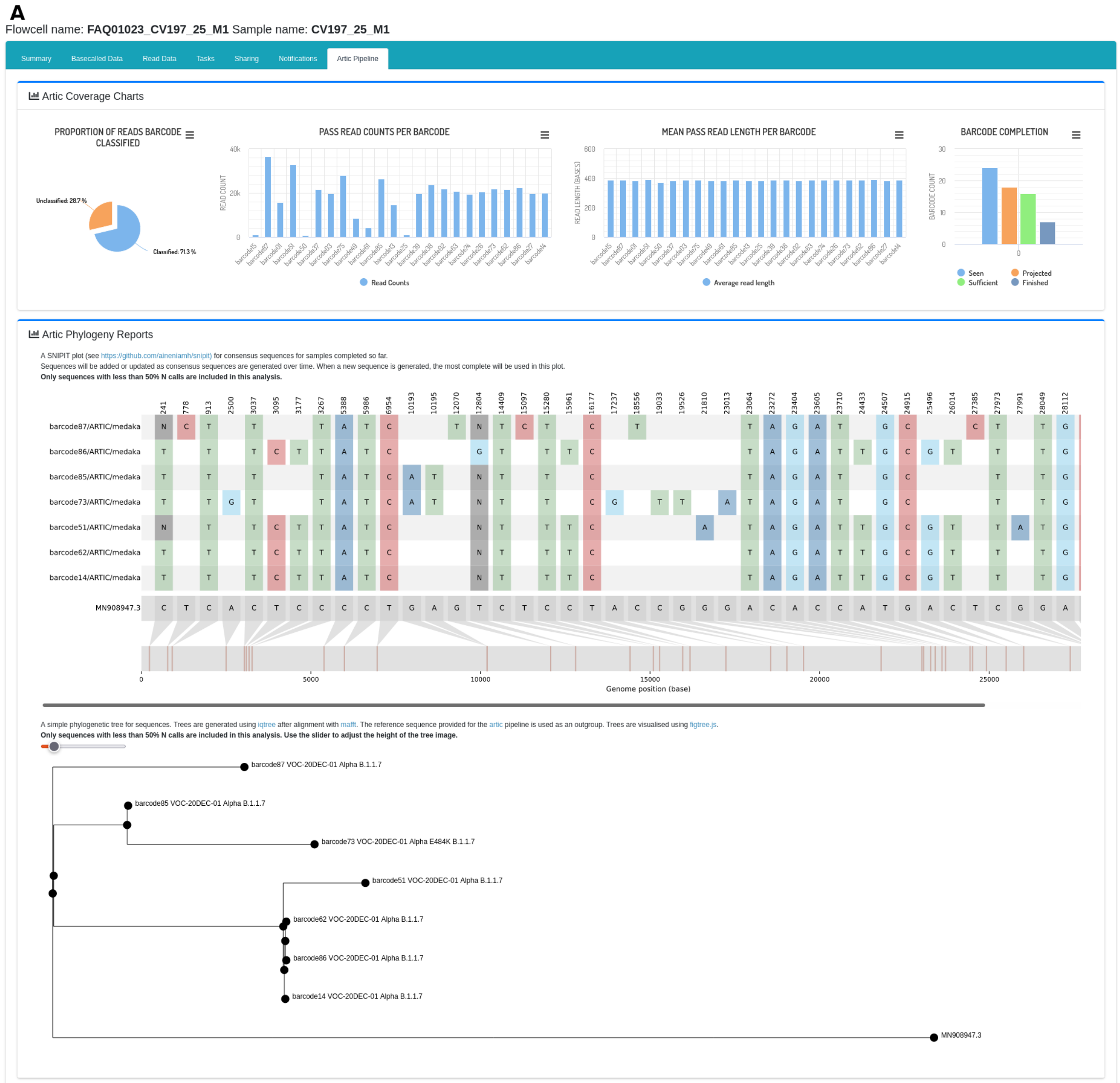

B

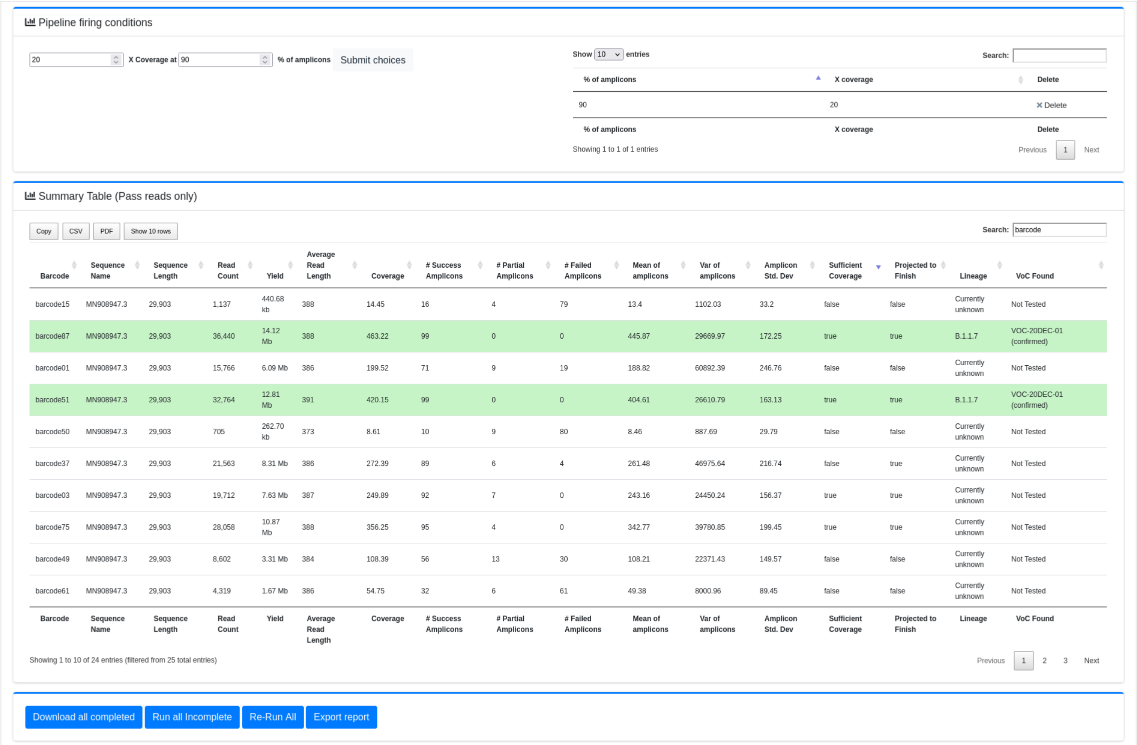

Pipeline firing conditions

20 X Coverage at 90 % of amplicons Submit choices

Show 10 entries Search:

% of amplicons	X coverage	Delete
90	20	✕ Delete

% of amplicons X coverage Delete

Showing 1 to 1 of 1 entries Previous 1 Next

Summary Table (Pass reads only)

Copy CSV PDF Show 10 rows Search: barcode

Barcode	Sequence Name	Sequence Length	Read Count	Yield	Average Read Length	Coverage	# Success Amplicons	# Partial Amplicons	# Failed Amplicons	Mean of amplicons	Var of amplicons	Amplicon Std. Dev	Sufficient Coverage	Projected to Finish	Lineage	VoC Found
barcode15	MN908947.3	29,903	1,137	440.68 kb	388	14.45	16	4	79	13.4	1102.03	33.2	false	false	Currently unknown	Not Tested
barcode87	MN908947.3	29,903	36,440	14.12 Mb	388	463.22	99	0	0	445.87	29669.97	172.25	true	true	B.1.1.7	VOC-20DEC-01 (confirmed)
barcode01	MN908947.3	29,903	15,766	6.09 Mb	386	199.52	71	9	19	188.82	60892.39	246.76	false	false	Currently unknown	Not Tested
barcode51	MN908947.3	29,903	32,764	12.81 Mb	391	420.15	99	0	0	404.61	26610.79	163.13	true	true	B.1.1.7	VOC-20DEC-01 (confirmed)
barcode50	MN908947.3	29,903	705	262.70 kb	373	8.61	10	9	80	8.46	887.69	29.79	false	false	Currently unknown	Not Tested
barcode37	MN908947.3	29,903	21,563	8.31 Mb	386	272.39	89	6	4	261.48	46975.64	216.74	false	true	Currently unknown	Not Tested
barcode03	MN908947.3	29,903	19,712	7.63 Mb	387	249.89	92	7	0	243.16	24450.24	156.37	true	true	Currently unknown	Not Tested
barcode75	MN908947.3	29,903	28,058	10.87 Mb	388	356.25	95	4	0	342.77	39780.85	199.45	true	true	Currently unknown	Not Tested
barcode49	MN908947.3	29,903	8,602	3.31 Mb	384	108.39	56	13	30	108.21	22371.43	149.57	false	false	Currently unknown	Not Tested
barcode61	MN908947.3	29,903	4,319	1.67 Mb	386	54.75	32	6	61	49.38	8000.96	89.45	false	false	Currently unknown	Not Tested

Barcode Sequence Name Sequence Length Read Count Yield Average Read Length Coverage # Success Amplicons # Partial Amplicons # Failed Amplicons Mean of amplicons Var of amplicons Amplicon Std. Dev Sufficient Coverage Projected to Finish Lineage VoC Found

Showing 1 to 10 of 24 entries (filtered from 25 total entries) Previous 1 2 3 Next

Download all completed Run all Incomplete Re-Run All Export report

C

Lineage Information for barcode87/ARTIC/medaka - B.1.1.7

Links to information on lineage B.1.1.7

PANGO Info | Outbreak Info

Variant of Concern Report for barcode87/ARTIC/medaka

If a genome is analysed, this will provide a report for the variant or variants found based on current PHE Variant Definitions. This analysis will report all possible VoCs and caution should be taken in interpretation of low coverage/quality genomes.

Reported VuI/VoCs:

sample_id	phe-label	unique-id	status	mutation-ref-calls	mutation-mixed-calls	mutation-calls	indel-ref-calls	indel-calls	no-calls	no-calls-deletion
barcode87/ARTIC/medaka	VOC-20DEC-01	denture-daughter	confirmed	0	0	13	0	2	0	0

Observed mutations:

type	snp	snp	snp	snp	snp	snp	snp	del	snp	snp	snp	snp	snp	snp	del	del
**reference-base**	T	C	C	C	C	C	T	GTCTGGTTTT	G	C	T	C	T	C	ATACATG	TTTA
**variant-base**	C	T	T	T	A	T	C	G	T	T	C	T	C	T	A	T
**var-length**	1	1	1	1	1	1	1	10	1	1	1	1	1	1	7	4
**one-based-reference-position**	778	913	3037	3267	5388	5986	6954	11287	12070	14408	15096	15279	16176	18555	21764	21990
**iupac-variant-bases**	C	T	T	T	A	T	C	G	T	T	C	T	C	T	A	T

type	snp	snp	snp	snp	snp	snp	snp	snp	snp	snp	snp	del	mnp	mnp-snp	mnp-snp
**reference-base**	A	C	A	C	C	T	G	T	C	G	A	TA	GAT	A	T
**variant-base**	T	A	G	A	T	G	C	C	T	T	G	T	CTA	T	A
**var-length**	1	1	1	1	1	1	1	1	1	1	1	2	3	1	1
**one-based-reference-position**	23063	23271	23403	23604	23709	24506	24914	27384	27972	28048	28111	28270	28280	28281	28282
**iupac-variant-bases**	T	A	G	A	T	G	C	C	T	T	G	T	CTA	T	A

Sample_ID: barcode87/ARTIC/medaka
PANGO:B.1.1.7
nextstrain:N501Y.V1

Variant Status: confirmed

PHE-Label: VOC-20DEC-01
WHO Label: Alpha
Alternate Names: VOC202012/01, UK variant, Kent variant, VOC1,

Description:
This variant became widespread in the UK in the Winter of 2021 and is characterised by increased transmissibility.
Information Sources Source_1 Source_2

Variant Calls Detected:

Position	gene	protein	ref	variant	sample call	type	status
3267	ORF1ab	nsp3	C	T	T	SNP	detect
5388	ORF1ab	nsp3	C	A	A	SNP	detect
6954	ORF1ab	nsp3	T	C	C	SNP	detect
21764	S	surface glycoprotein	ATACATG	A	A	deletion	detect
21990	S	surface glycoprotein	TTTA	T	T	deletion	detect
23063	S	surface glycoprotein	A	T	T	SNP	detect
23271	S	surface glycoprotein	C	A	A	SNP	detect
23604	S	surface glycoprotein	C	A	A	SNP	detect
23709	S	surface glycoprotein	C	T	T	SNP	detect
24506	S	surface glycoprotein	T	G	G	SNP	detect
24914	S	surface glycoprotein	G	C	C	SNP	detect
27972	ORF8	ORF8 protein	C	T	T	SNP	detect
28048	ORF8	ORF8 protein	G	T	T	SNP	detect
28111	ORF8	ORF8 protein	A	G	G	SNP	detect
28280	N	nucleocapsid phosphoprotein	GAT	CTA	CTA	MNP	detect

One based position - notes on sites detected in this sample.

The observed calls were:

- mutation_ref_calls: 0
- indel_ref_calls: 0
- mutation_calls: 13
- mutation_mixed_calls: 0
- indel_calls: 2
- no_calls: 0
- no_call_deletion: 0

Classification rules:

confirmed
- mutations_required: 13
- indels_required: 0
- allowed_wildtype: 0

probable
- mutations_required: 5
- indels_required: 0
- allowed_wildtype: 0

low_qc
- mutations_required: 0
- indels_required: 0
- allowed_wildtype: 0

Acknowledgements:
*Curators:*Natalie Groves Ulf Schaefer Nick Loman

The complete list of variants of concern scanned included:
VUI-21FEB-04 VOC-20DEC-02 VUI-21FEB-01 VUI-21MAR-01 VUI-21MAY-01 VOC-20DEC-01 VOC-21APR-02 VUI-21FEB-03 VUI-21APR-01 E484K VUI-21JUL-01 VUI-21JAN-01 VUI-21MAR-02 VUI-21MAY-02 VUI-21JUN-01 VOC-21JAN-02 VUI-21APR-03 VOC-21FEB-02

D

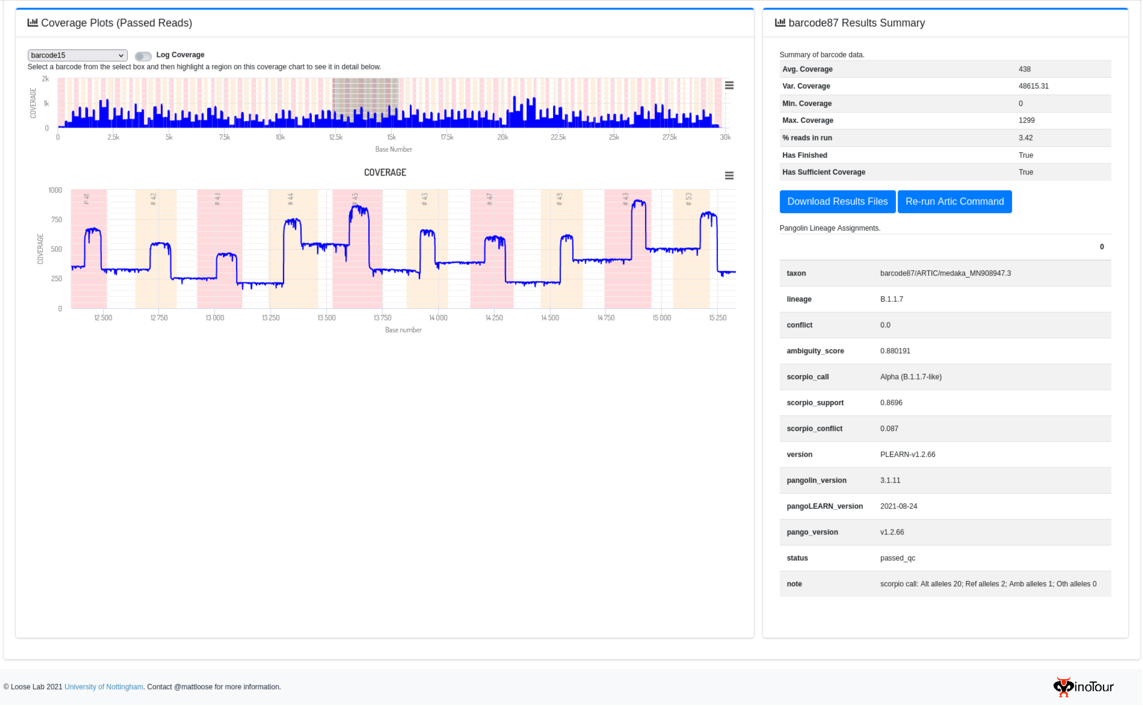

Supplementary Figure 1: Screenshot of the ARTIC tab in minoTour. A) The top section of the tab deals with visualising overall run metrics, including the proportion of mapped reads classified as a barcode in a run, the read count per barcode, the mean read length and the total number of amplicons that have over 20X coverage, over 1X coverage and 0X coverage. All generated consensus sequences have a SNIPIT report created for them, and are added to a growing phylogenetic tree created for the run. B) The next section displays the conditions required for a sample to run the ARTIC medaka pipeline on it's accumulated reads. The default for a sample to be analysed is displayed, with 90% of amplicons on the sample at 20X. A sortable and searchable summary table displays information about each sample currently identified in the run. Samples that have been analysed are colour coded as green rows. C) This section deals with information about a specific sample chosen from the table in B) by clicking on it. If the sample has sufficient data and the medaka pipeline has been run, a report generated by the aln2type tool is displayed, confirming lineage and SNPs seen in the consensus. D). The coverage is displayed for the genome selected from the table in B), along with the ARTIC lineage report.

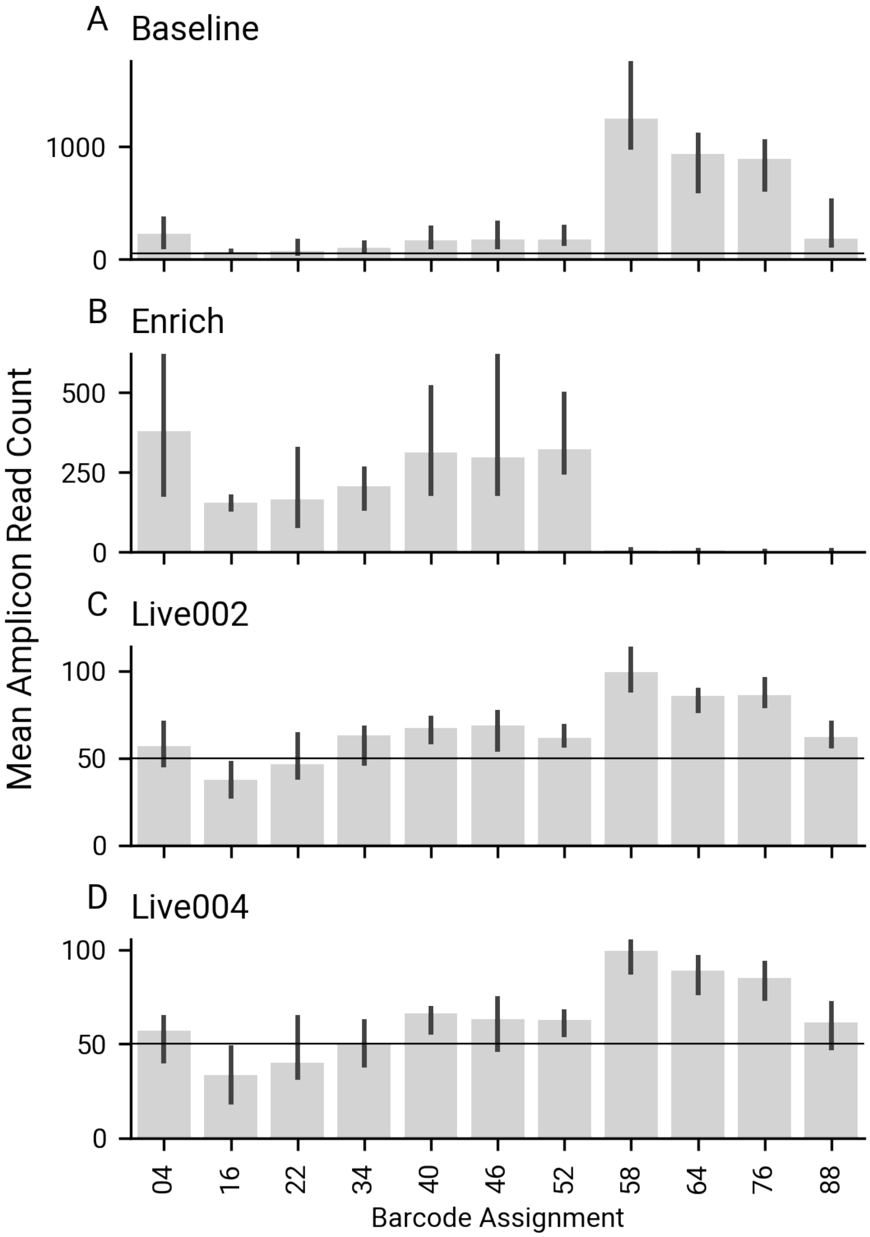

Supplementary Figure 2: Barcode aware adaptive sampling applied to 1,200 base pair SARS-CoV-2 amplicons. 11 SARS-CoV-2 samples which generated sequence data in a single sequencing library were sequenced under different conditions. A) With no adaptive sampling, the library is clearly over abundant with respect to barcodes 58,64,76 and 88. B) The same library was sequenced with barcodes 58,64,76 and 88 depleted entirely using ReadFish. C) The rejection strategy was switched from a simple list of barcodes to using Swordfish to monitor coverage of each barcode/amplicon combination in real-time. The majority of over abundant barcodes are rapidly switched off. Final coverage is more uniform. D) Repeat of the experiment illustrated in C.

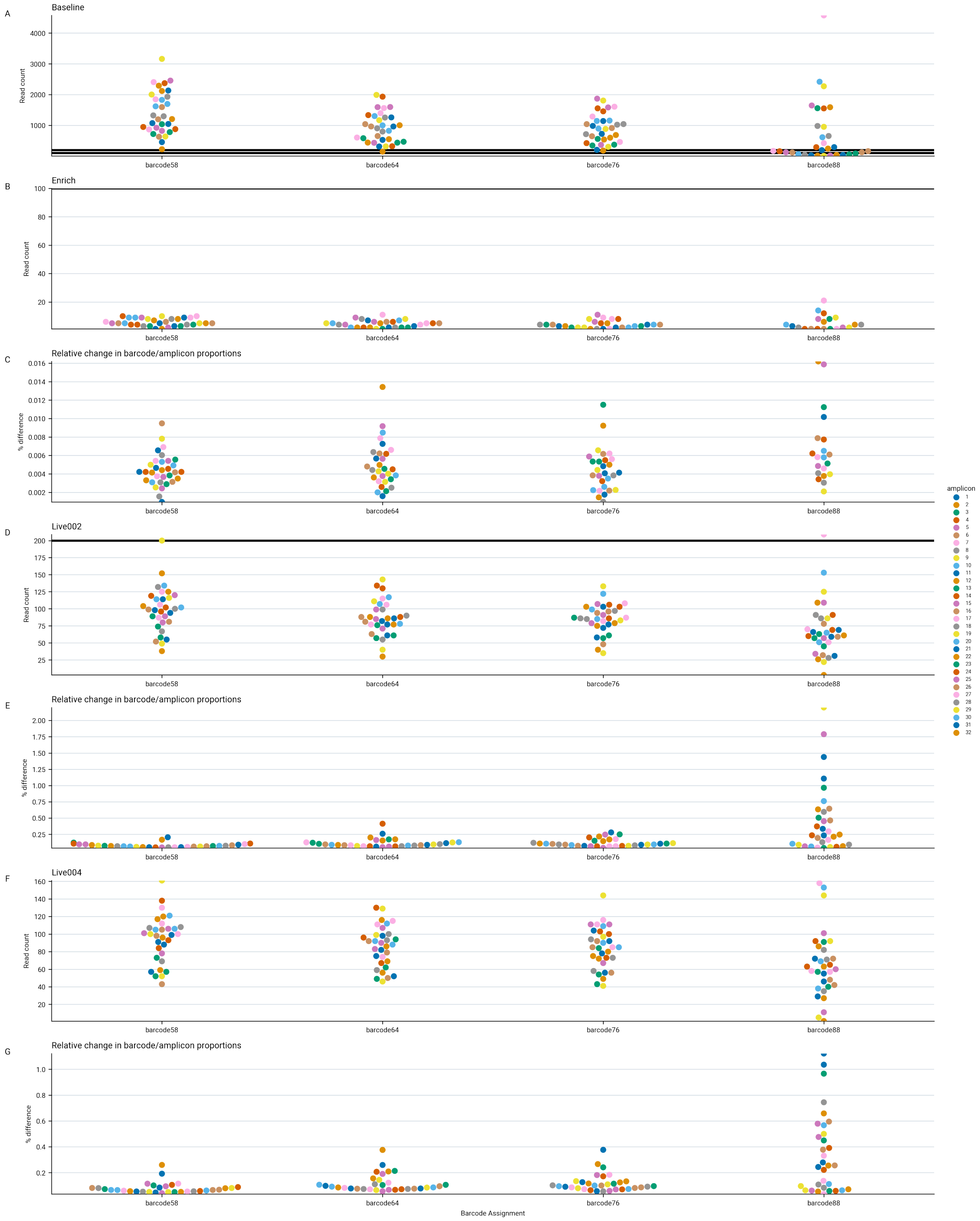

(Caption on next page.)

Supplementary Figure 3: Barcode aware adaptive sampling applied to 1200 base amplicon SARS-CoV-2. Of 11 SARS-CoV-2 samples, 4 illustratively overabundant barcodes were chosen to demonstrate the shift in amplicon coverages across different run conditions. A) with no adaptive sampling, the library is clearly over abundant with respect to barcodes 58, 64, 76 and 88. B,C) The same library was sequenced with barcodes 58,64,76 and 88 switched off using ReadFish. The relative change in proportion of data from each barcode/amplicon combination changes as a consequence (C). D,E) The rejection strategy was switched from a simple list of barcodes to using Swordfish to monitor coverage of each barcode/amplicon combination in real-time. The majority of over abundant barcodes are rapidly switched off. Some of the more under abundant amplicons are enriched in barcode88. Final coverage is more uniform. F,G) Repeat of the experiment illustrated in D,E.

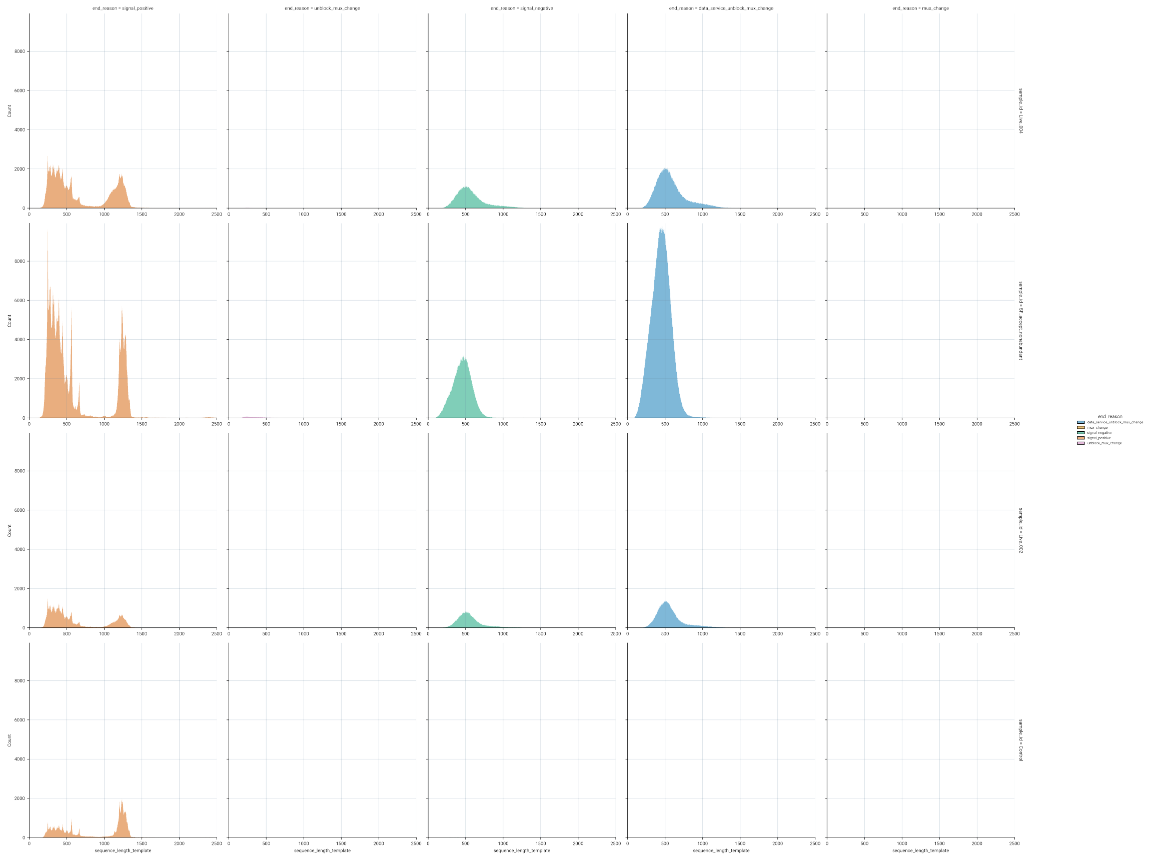

Supplementary Figure 4: Count of read sequence lengths split by end reasons for each run. Runs are Live004, enrich, Live002, Control, top to bottom. End reasons are derived from the sequencing summary files generated during the run. *Signal positive* represents a read sequencing naturally; *signal negative* is a read ending with the opposite polarity, most likely ending sequencing naturally; *unblock mux change* is a read unblocked during a mux scan; *mux_change* is a read ending and the channel switching to a new pore; *data_service_unblock_mux_change* represents an unblock signal being sent by the user to remove a read from further sequencing.

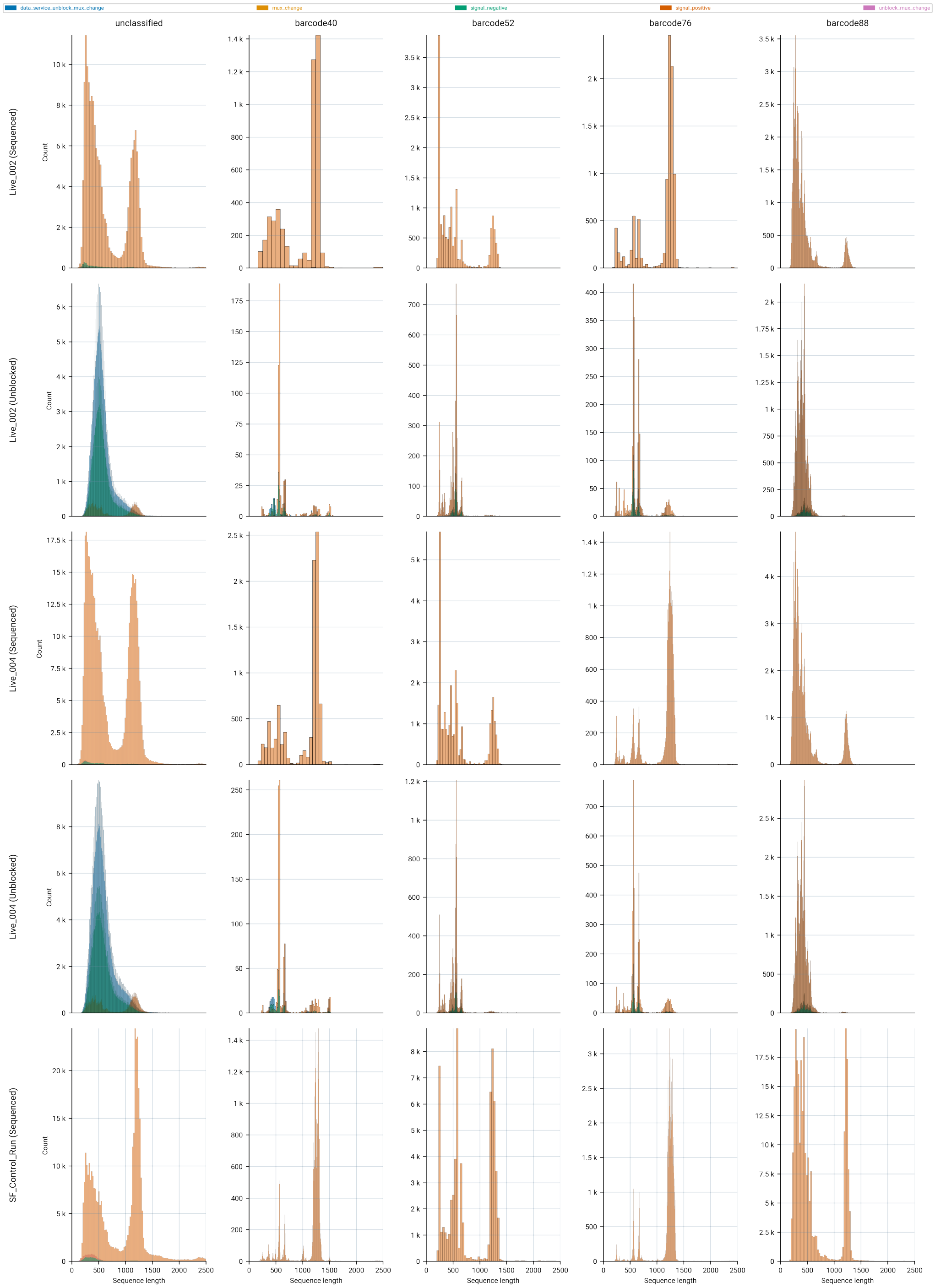

(Caption on next page.)

Supplementary Figure 5: Count of sequence lengths of reads from Illustrative barcodes (unclassified, barcode40, barcode52, barcode76, barcode88) in 4 runs (Control, Live002, Live004, enrich), split by read end reason, between unblocked and sequenced reads. End reasons are derived from the sequencing summary files generated during the run. *Signal positive* represents a read sequencing naturally; *signal negative* is a read ending with the opposite polarity, most likely ending sequencing naturally; *unblock mux change* is a read unblocked during a mux scan; *mux_change* is a read ending and the channel switching to a new pore; *data_service_unblock_mux_change* represents an unblock signal being sent by the user to remove a read from further sequencing. Enrich represents the run where overabundant barcodes were depleted, Live002 and Live004 represent runs with swordfish enabled with a coverage threshold of 50.

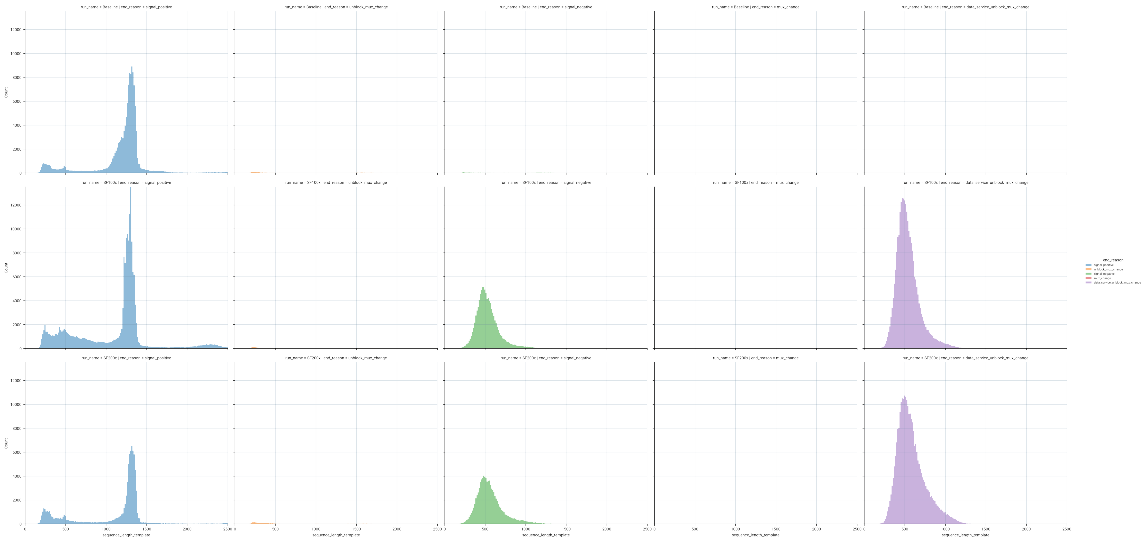

Supplementary Figure 6: Count of read sequence lengths split by end reasons for each run in our second experiment. Runs are Control, SF100, SF200, top to bottom. End reasons are derived from the sequencing summary files generated during the run. *Signal positive* represents a read sequencing naturally; *signal negative* is a read ending with the opposite polarity, most likely ending sequencing naturally; *unblock mux change* is a read unblocked during a mux scan; *mux_change* is a read ending and the channel switching to a new pore; *data_service_unblock_mux_change* represents an unblock signal being sent by the user to remove a read from further sequencing.

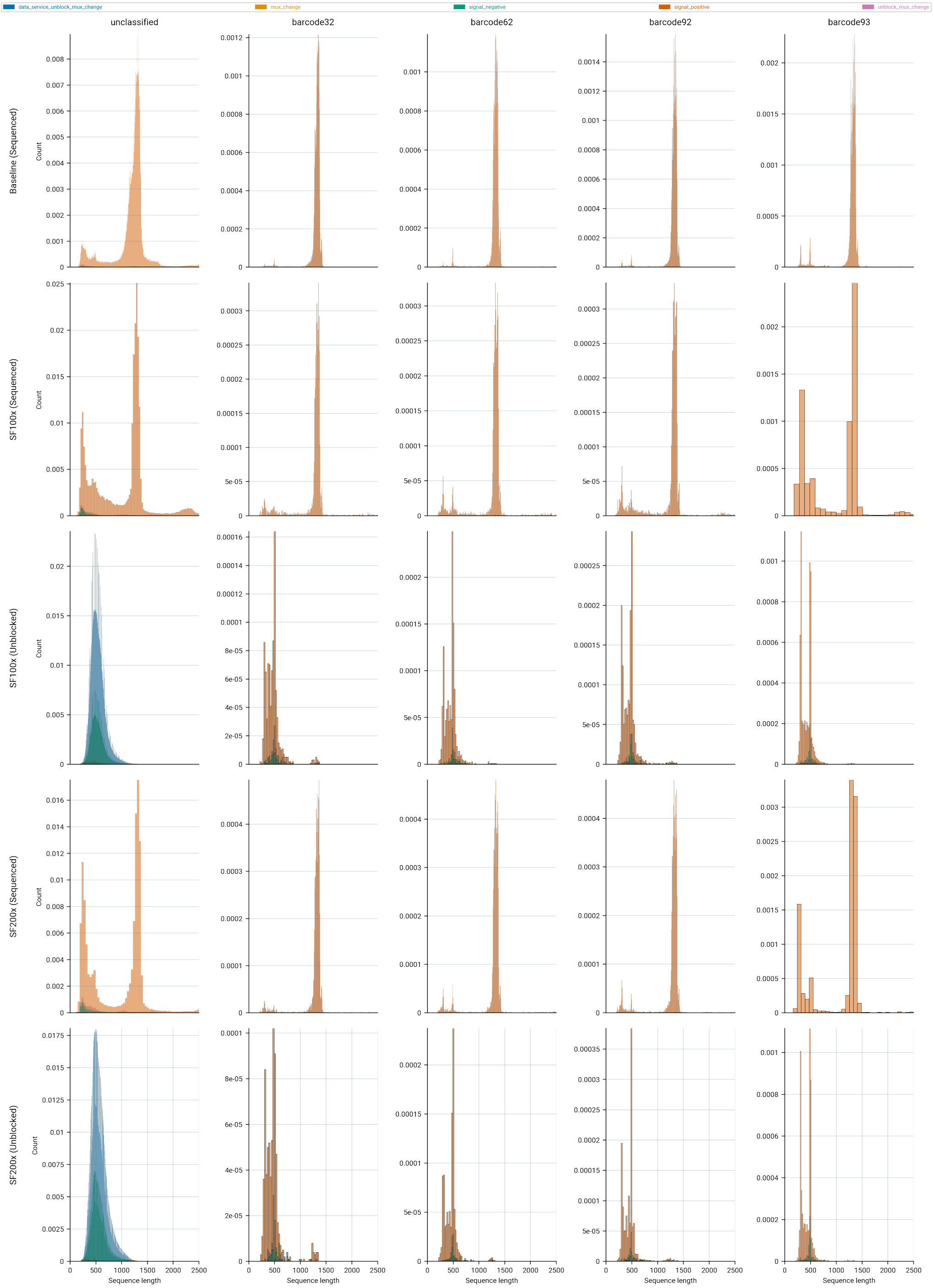

(Caption on next page.)

Supplementary Figure 7: Count of sequence lengths of reads from Illustrative barcodes (unclassified, 32, 62, 92, 93) in 3 runs (Control, SF100, SF200), split by read end reason, between unblocked and sequenced reads. End reasons are derived from the sequencing summary files generated during the run. *Signal positive* represents a read sequencing naturally; *signal negative* is a read ending with the opposite polarity, most likely ending sequencing naturally; *unblock mux change* is a read unblocked during a mux scan; *mux_change* is a read ending and the channel switching to a new pore; *data_service_unblock_mux_change* represents an unblock signal being sent by the user to remove a read from further sequencing. Enrich represents the run where overabundant barcodes were depleted, SF100 and SF200 represent runs with swordfish enabled with a coverage threshold of 100 and 200 respectively.

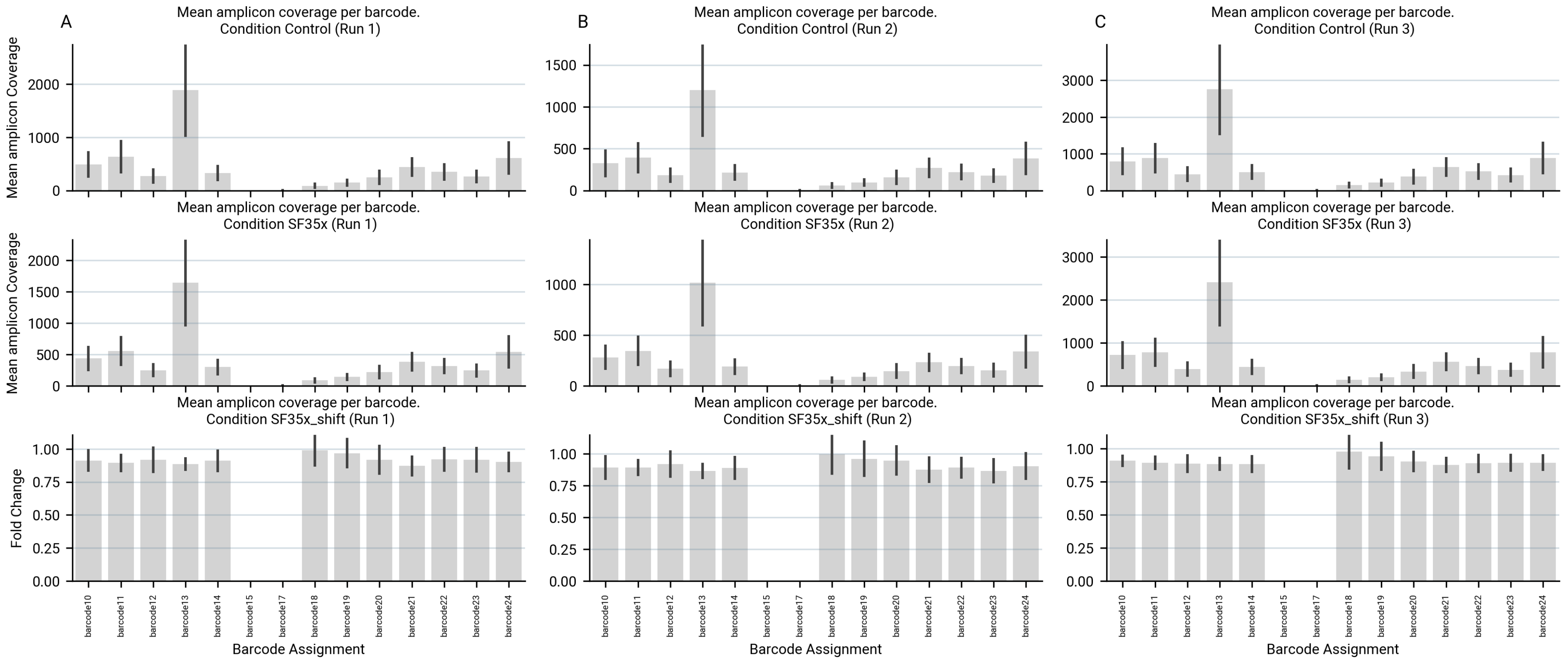

Supplementary Figure 8: The mean amplicon coverage for each barcode, for a library prepared using the midnight protocol. **A**) The first clinical data midnight protocol run. The first row displays the control condition, where no adaptive sampling was applied. This was the condition present for the odd numbered channels on a standard MinION flow cell. Barcode 13 is clearly overabundant. The second row displays the data for channels where adaptive sampling was enabled. Amplicons on any barcode which had achieved 35x coverage were unblocked. This was the condition present on the even numbered channels on a standard MinION flow cell. The bottom row displays the fold change for the mean amplicon coverage per barcode, between adaptive sampling enabled channels and control channels . Barcodes 15 and 17 are negative controls, with one read mapping to barcode 17. This has been removed for clarity on the plot. **B**) As A), but for a repeated second run. **C**) As A), but for a repeated third run.

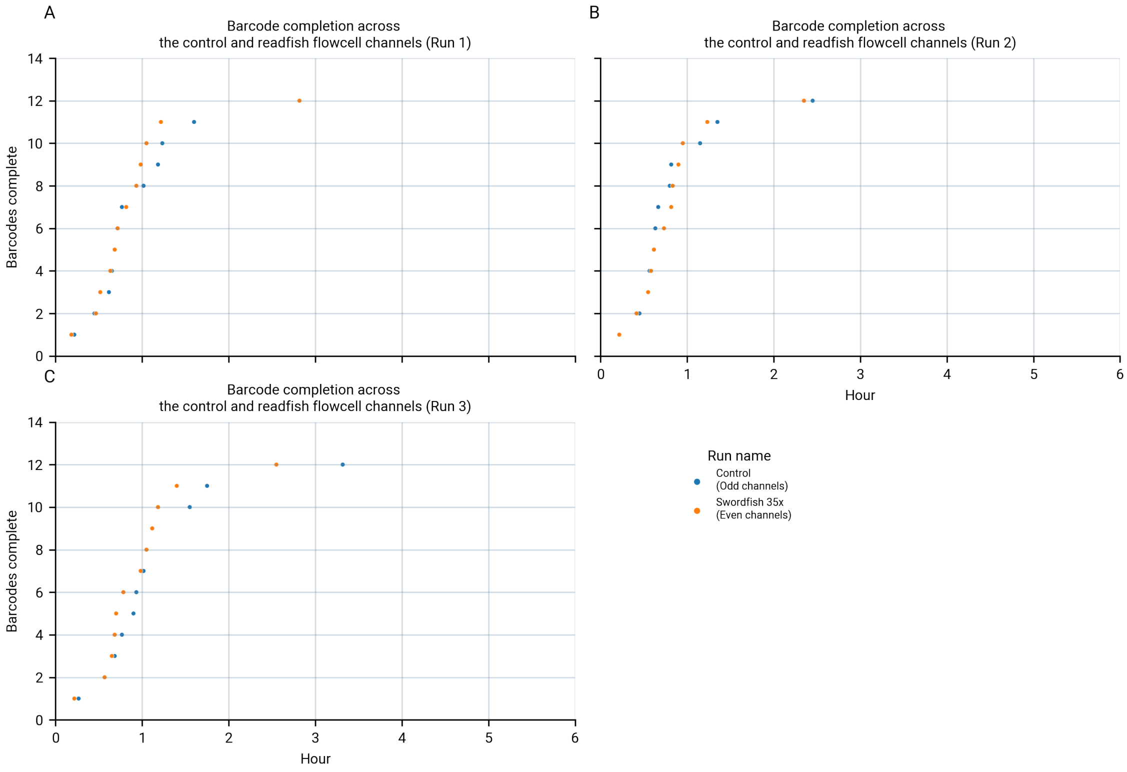

Supplementary Figure 9: The mean amplicon coverage for each barcode, for a library prepared using the midnight protocol. **A**) The time at which a barcode reached 90 % of amplicons at 20× for data produced either by the control channels (odd numbered), with no adaptive sampling enabled, or the adaptive sampling enabled channels (even numbered), for the first clinical data midnight protocol run. Adaptive sampling channels unblocked reads from amplicons that had achieved a 35× target coverage. Time is considered to have been saved if the barcode complete count is shifted to the left of the other condition. Any points above the control condition (at the same time) are considered as extra barcodes recovered. **B**) As A), but for a second repeated run. **C**) As B) but for a third repeated run.

2 Supplementary tables

Sample ID	Sample type	E gene Ct	S gene Ct	in house PCR	PCR cycles	Conc. ng/ul	Amount DNA for ideally 100 ng	Amount H2O	Barcode	Detected in Library
clin-001	swab/cDNA	Positive	12.31	33.54	1200 - 65°C	14.9	7	3	Barcode88	Yes
clin-002	swab/cDNA	26.99	26.69	23.31	1200 - 65°C	34.8	3	7	Barcode76	Yes
clin-003	swab/cDNA	27.48	25.19	31.92	1200 - 65°C	51.6	2	8	Barcode64	Yes
clin-004	swab/cDNA	16.5	18.4		1200 - 65°C	4.3	10	0	Barcode52	Yes
clin-005	swab/cDNA	23.3	25.4		1200 - 65°C	3.08	10	0	Barcode40	Yes
clin-006	swab/cDNA	30.5	32.2		1200 - 65°C	0.264	10	0	Barcode28	Not detected
clin-007	swab/cDNA	21.3	23.2		1200 - 65°C	too low	10	0	Barcode16	Yes (single amplicon)
clin-008	swab/cDNA	14.41	unknown		1200 - 65°C	3.74	10	0	Barcode04	Yes
clin-009	swab/cDNA	21.15	unknown		1200 - 65°C	0.46	10	0	Barcode94	Not detected
clin-010	swab/cDNA	19	unknown		1200 - 65°C	0.114	10	0	Barcode82	Not detected
clin-011	swab/cDNA	25	unknown		1200 - 65°C	0.394	10	0	Barcode70	Not detected
clin-012	swab/cDNA	unknown	unknown		1200 - 65°C	90.8	2	8	Barcode58	Yes
clin-013	swab/cDNA	unknown	unknown	25	1200 - 65°C	2.9	10	0	Barcode46	Yes
Positive Control 1	culture/cDNA				1200 - 65°C	2	10	0	Barcode34	Yes
Positive Control 2	culture/cDNA				1200 - 65°C	1.05	10	0	Barcode22	Yes
Negative Control					1200 - 65°C	too low	10	0	Barcode10	Not detected (Correct)

Supplementary Table 1: CT values and sample information for the SARS-CoV-2 samples that were used in the first experiment.

	**Total Amplicons Seen**		**Amplicons from non-abundant barcodes only**	
	**>0X**	**>50X**	**>0X**	**>50X**
Control Run	306	271	178	150
Deplete Abundant	287	166	178	166
Live 002	305	222	177	109
Live 004	306	214	178	103

Supplementary Table 2: Total count of barcode/amplicons seen in each experiment, and count of amplicons over 50x Coverage, after 110 minutes of sequencing.

Run	Yield (Basepairs)	Time
SF Control Run	388,212,762	110 Minutes
SF accept nonabundant	422,515,038	110 Minutes
Live 002	434,545,437	110 Minutes
Live 004	314,024,726	110 Minutes
SF200x	2,358,637,912	6 Hours
SF100x	2,306,728,845	6 Hours
Baseline	1,963,833,152	6 Hours

Supplementary Table 3: Run yields during the given time period for all experiment runs.

	Total amplicons seen	Amplicons over 50x
Control Run	2,736	2,478
Swordfish 100x	2,763	2,579
Swordfish 200x	2,753	2,586

Supplementary Table 4: Total count of barcode/amplicons seen in our second experiment, and those over 50x Coverage, after 360 minutes of sequencing.

3 Supplementary files

3.1 Supplementary file 1

PDF reports generated by the ARTIC tab in minoTour, bundled and compressed into a tar.gz file. Each barcode has a report generated, showing a simple line chart of coverage along the genome, and if the barcode has been run through the ARTIC medaka pipeline, the aln2type output report is included. A PDF summary for the whole run is also included, containing the mean read length and count per barcode, the SNIPIT plot of the run and the phylogenetic tree.

3.2 Supplementary file 2

CSV of lineage assigned to each sample at the FR, RU and SA timepoints for both the medaka and nanopolish generated consensus sequences. Cells marked N/A did not have sequences for that time point for both medaka and nanopolish, and cells marked None did not generate a lineage after running pangolin on them.

3.3 Supplementary file 3

Output CSV of nextclade (https://clades.nextstrain.org) web app for all our medaka consensus sequences.

3.4 Supplementary file 4

Output CSV of nextclade (https://clades.nextstrain.org) web app for all our nanopolish consensus sequences.

3.5 Supplementary file 5

Bed file provided for the custom 1200 amplicon scheme.
