# Supplementary figures and images for: A framework for real-time monitoring, analysis and adaptive sampling of viral amplicon nanopore sequencing
Source: Front Genet. 2023 Mar 27;14:1138582. doi: 10.3389/fgene.2023.1138582 (PMC10083257; doi:10.3389/fgene.2023.1138582)

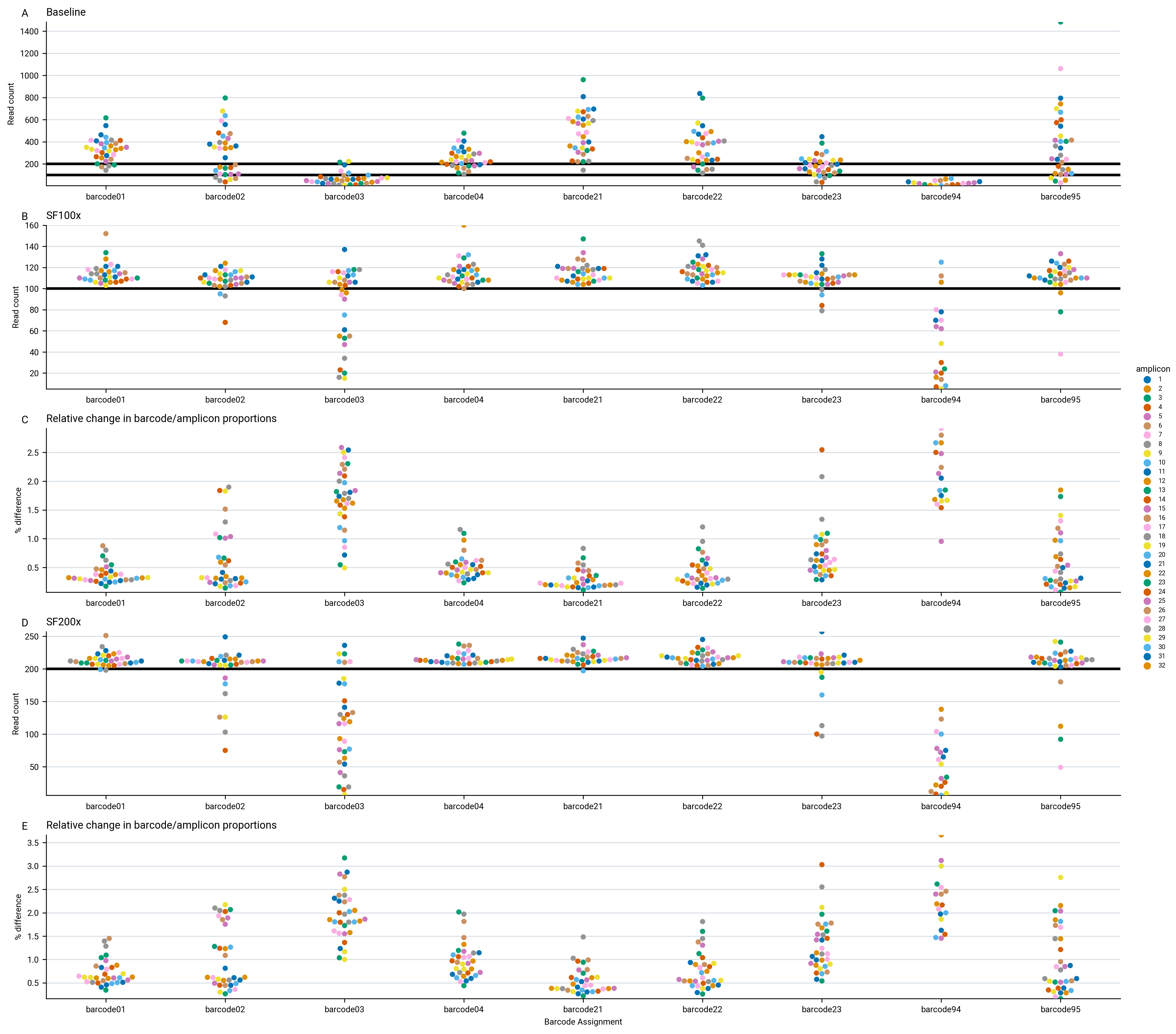

Supplement: Supplementary file 1 [file DataSheet1.ZIP › supplementary_data/Supplementary Info tex/Figures/sup_figure_3.png]

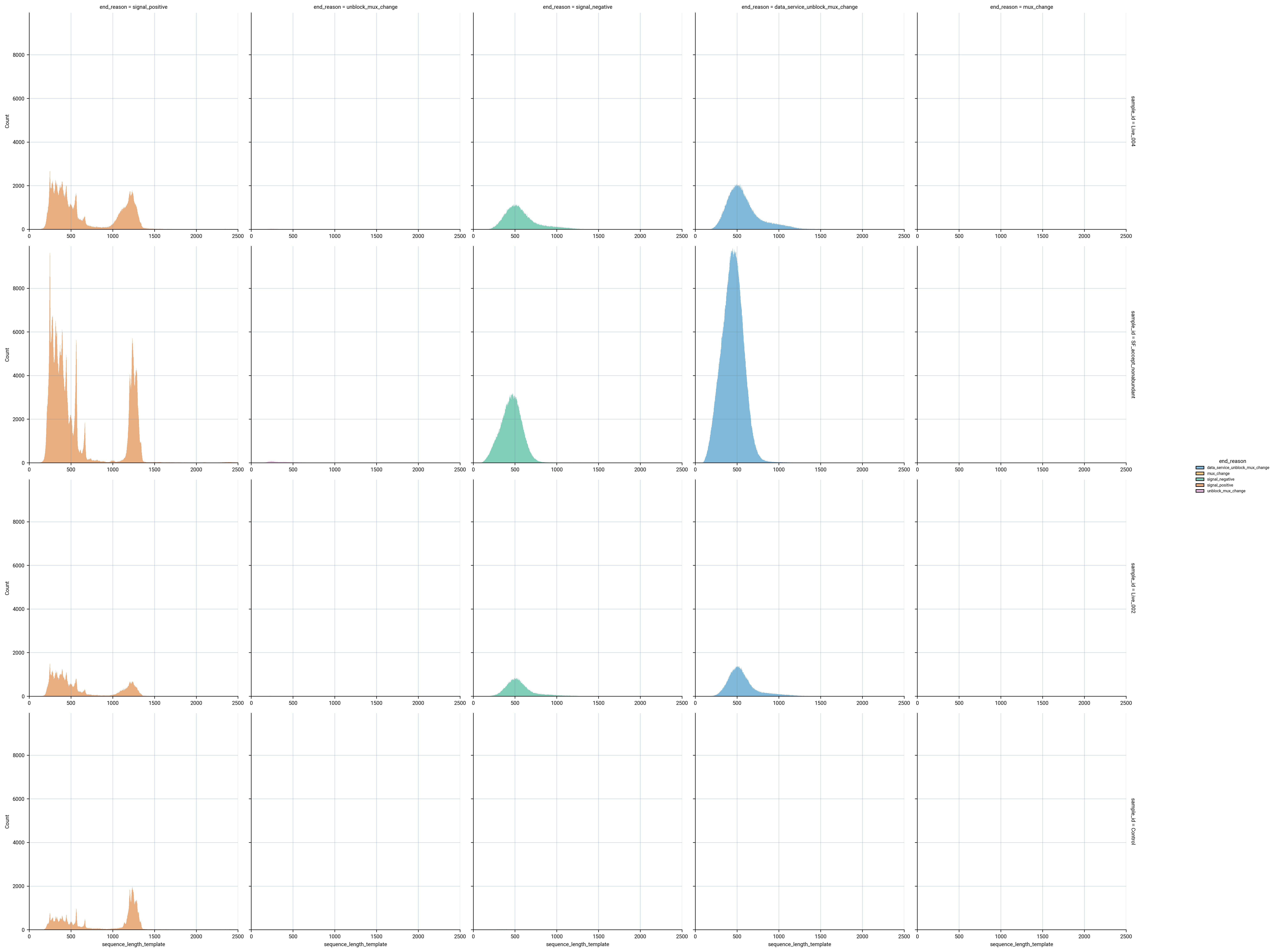

Supplement: Supplementary file 1 [file DataSheet1.ZIP › supplementary_data/Supplementary Info tex/Figures/sup_figure_4.png]

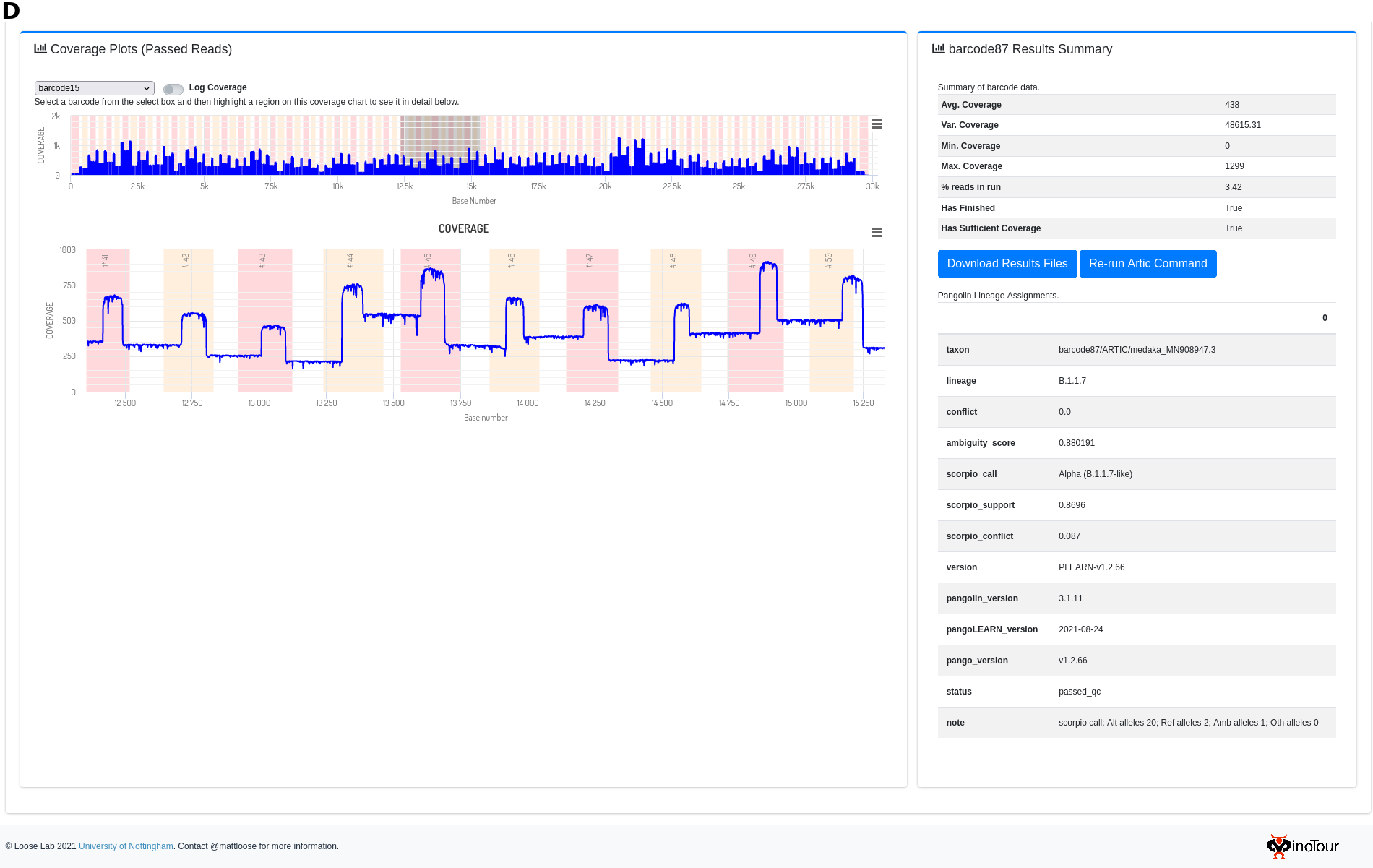

Supplement: Supplementary file 1 [file DataSheet1.ZIP › supplementary_data/Supplementary Info tex/Figures/coverage_plot_crop.png]

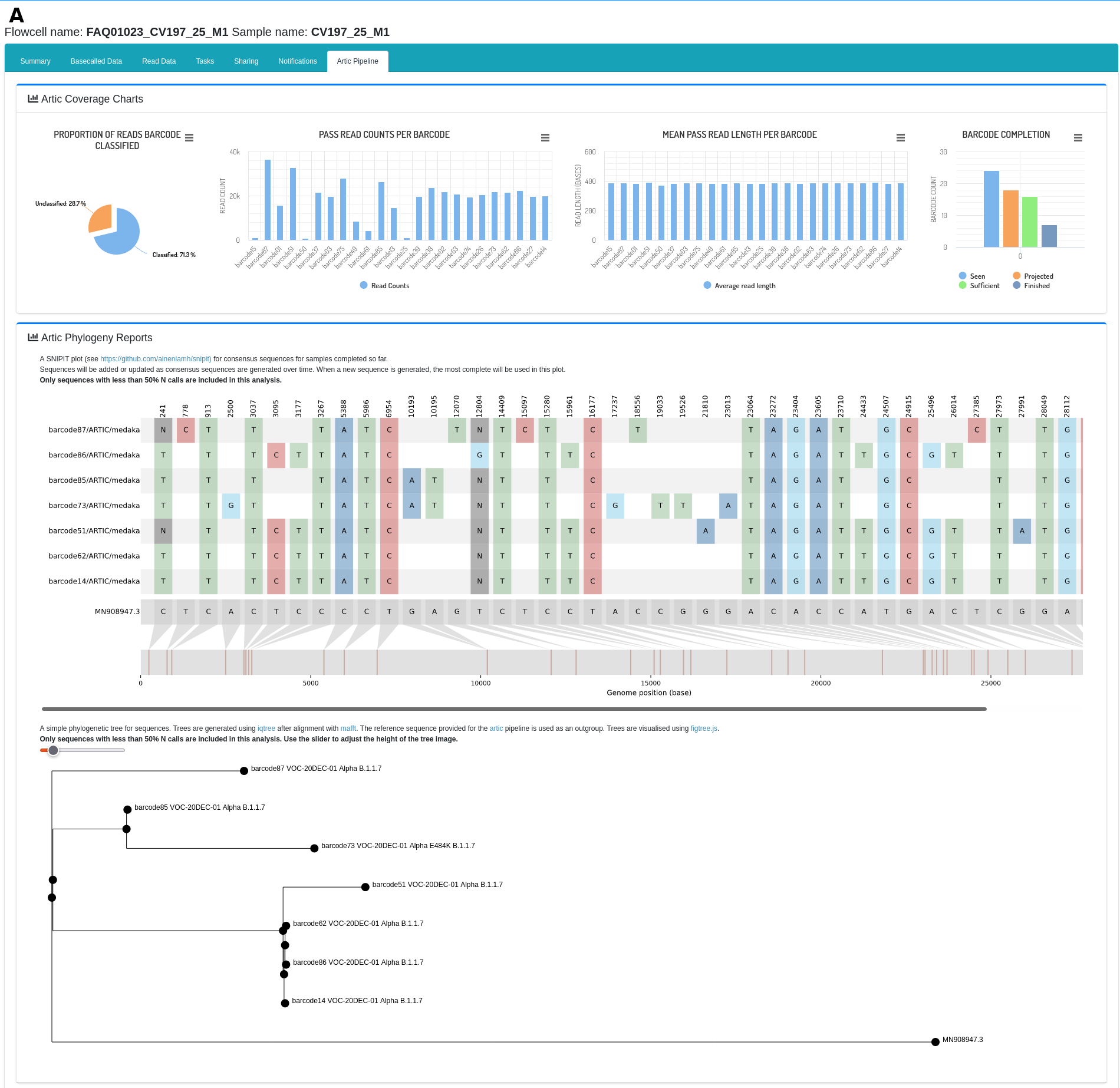

Supplement: Supplementary file 1 [file DataSheet1.ZIP › supplementary_data/Supplementary Info tex/Figures/phylogeney_crop.png]

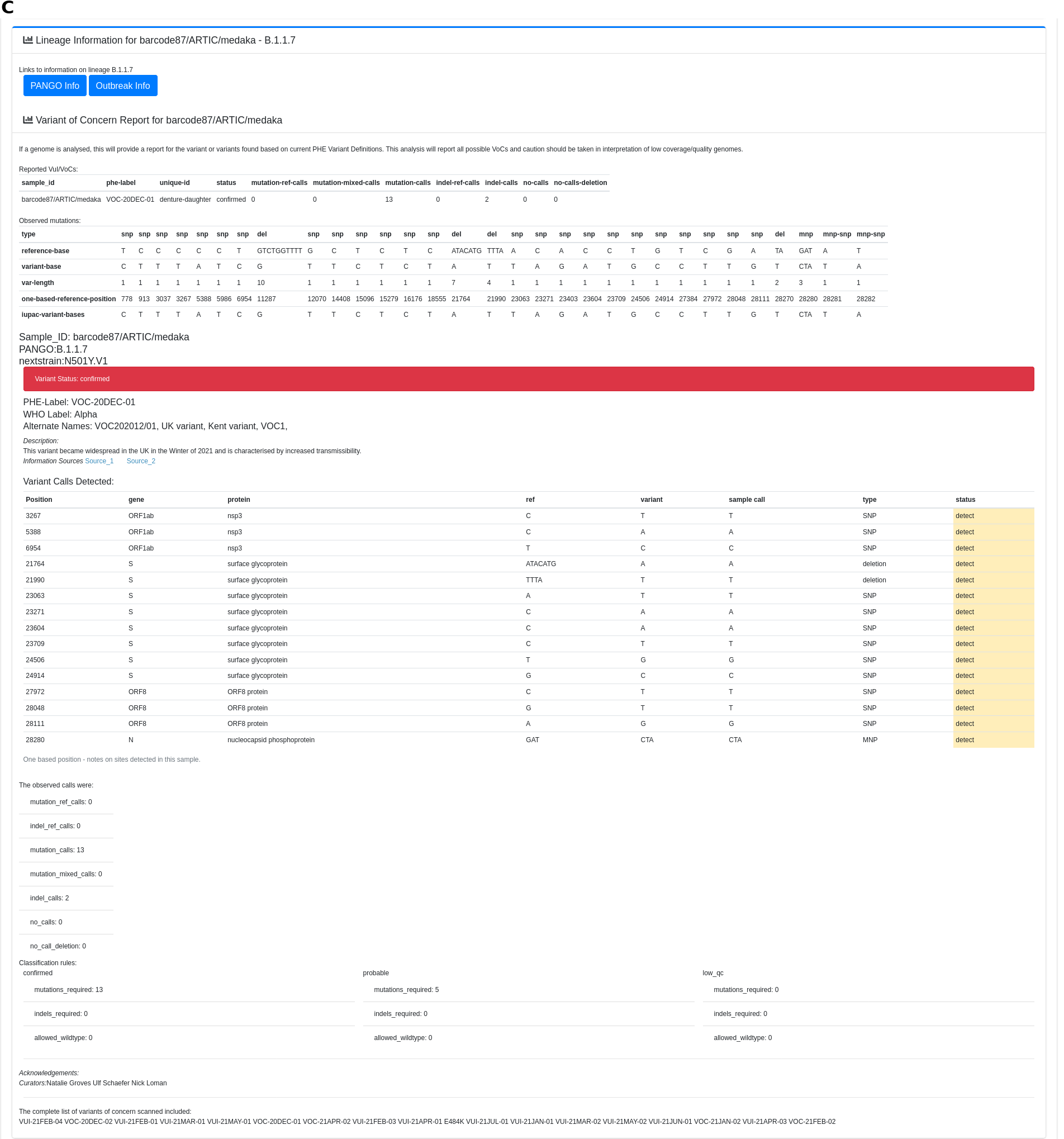

Supplement: Supplementary file 1 [file DataSheet1.ZIP › supplementary_data/Supplementary Info tex/Figures/snp_crop.png]

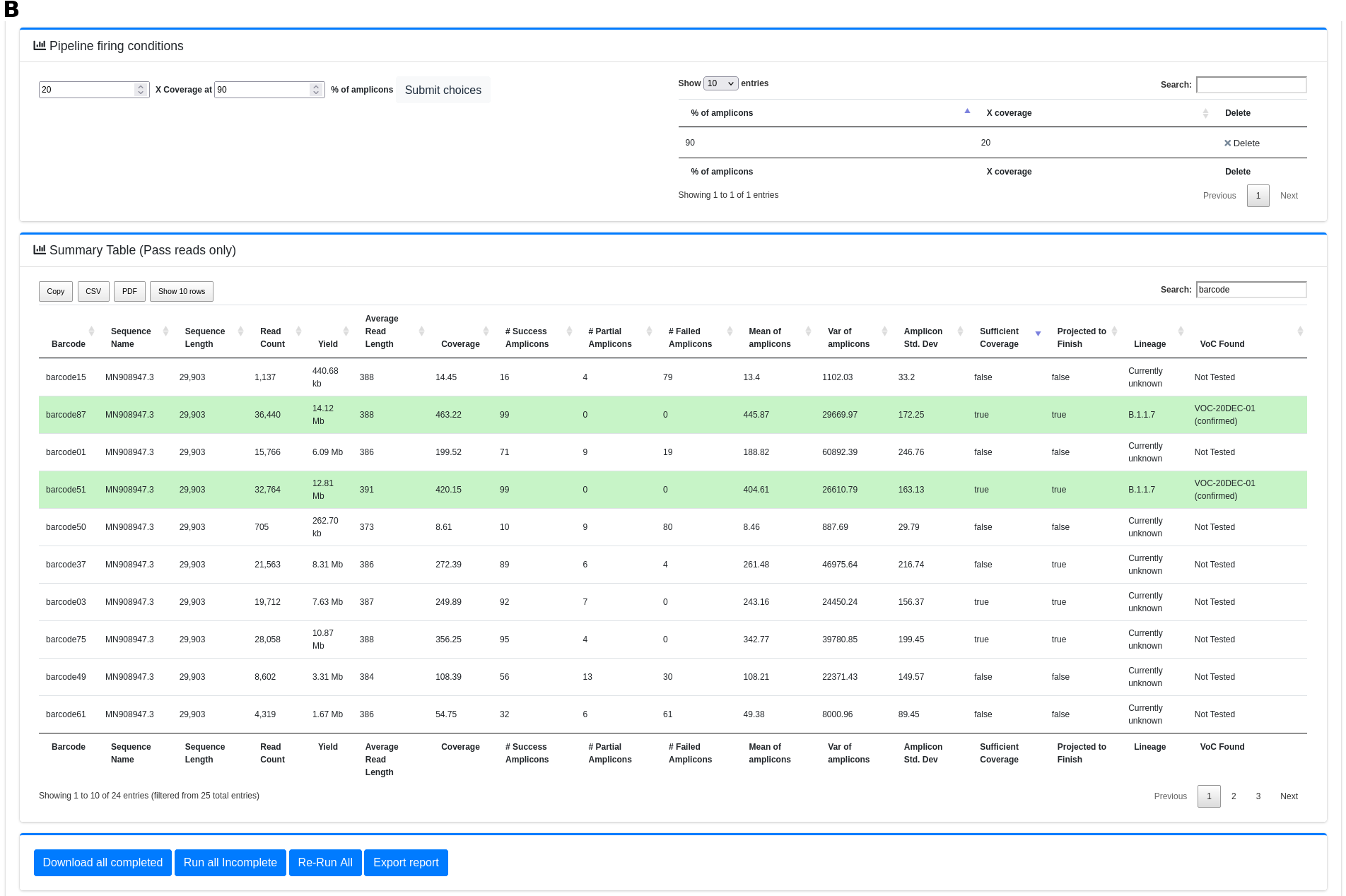

Supplement: Supplementary file 1 [file DataSheet1.ZIP › supplementary_data/Supplementary Info tex/Figures/summary_table_crop.png]

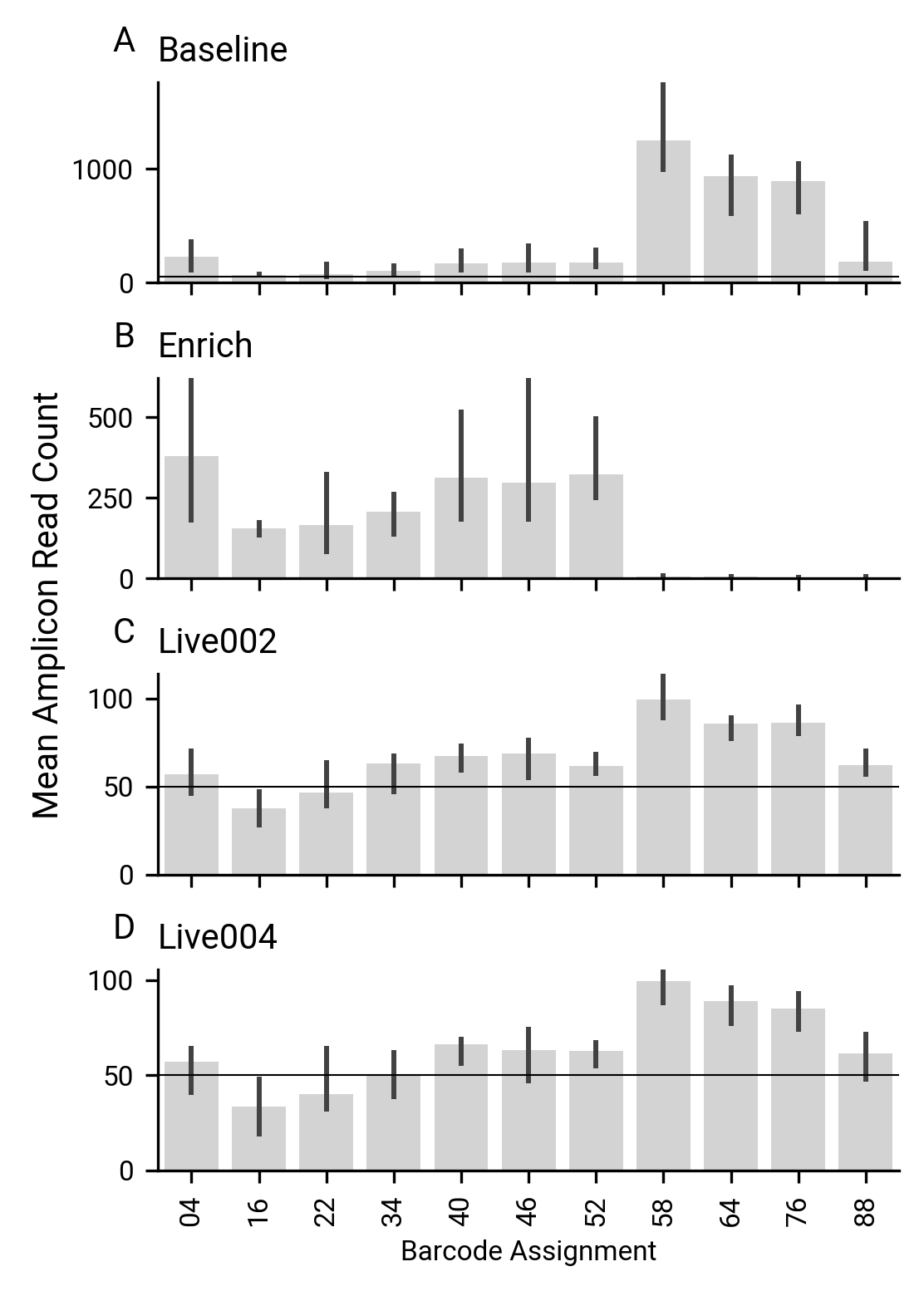

Supplement: Supplementary file 1 [file DataSheet1.ZIP › supplementary_data/Supplementary Info tex/Figures/our_runs_read_counts_per_barcode.png]

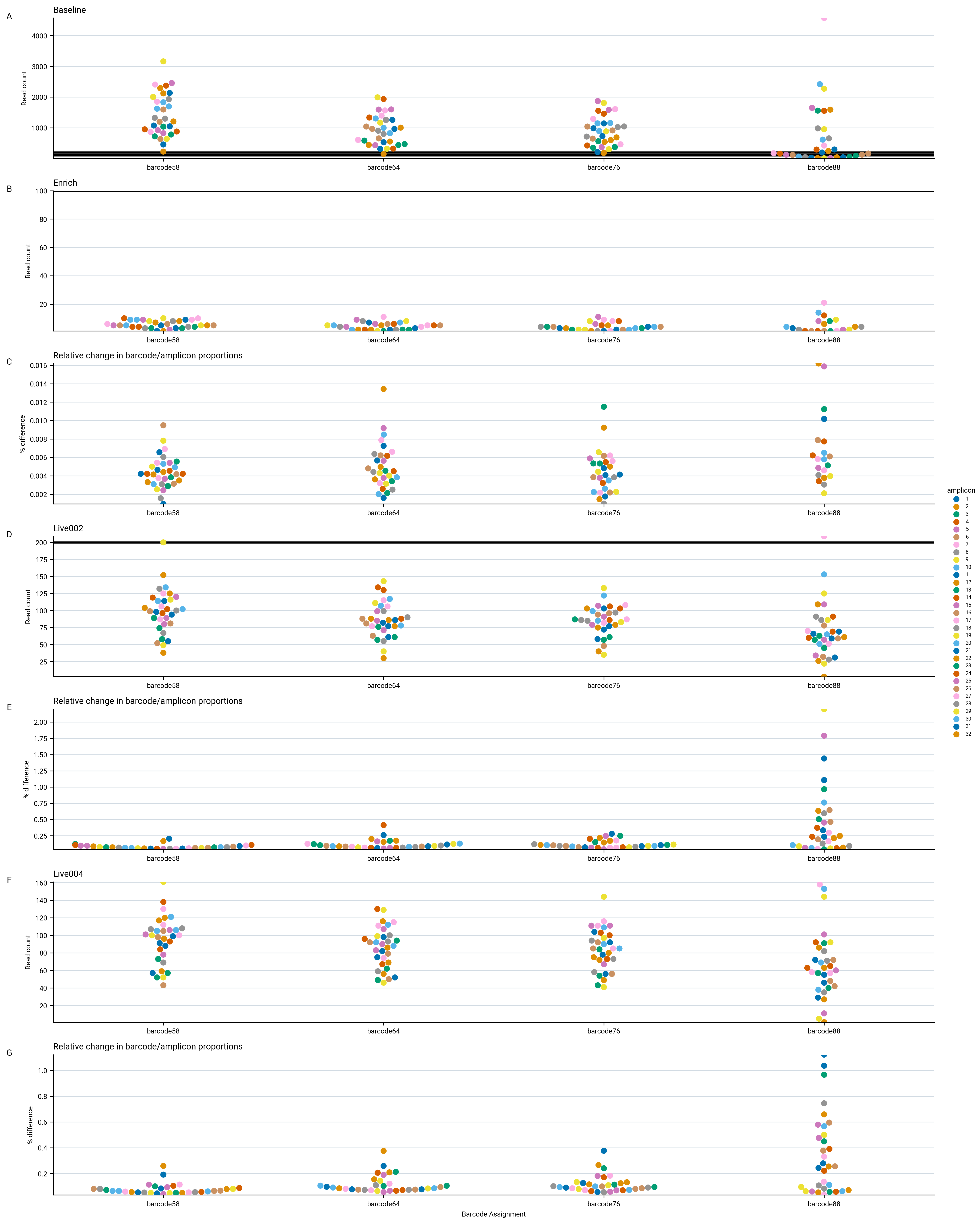

Supplement: Supplementary file 1 [file DataSheet1.ZIP › supplementary_data/Supplementary Info tex/Figures/our_runs_illustrative_barcodes.png]

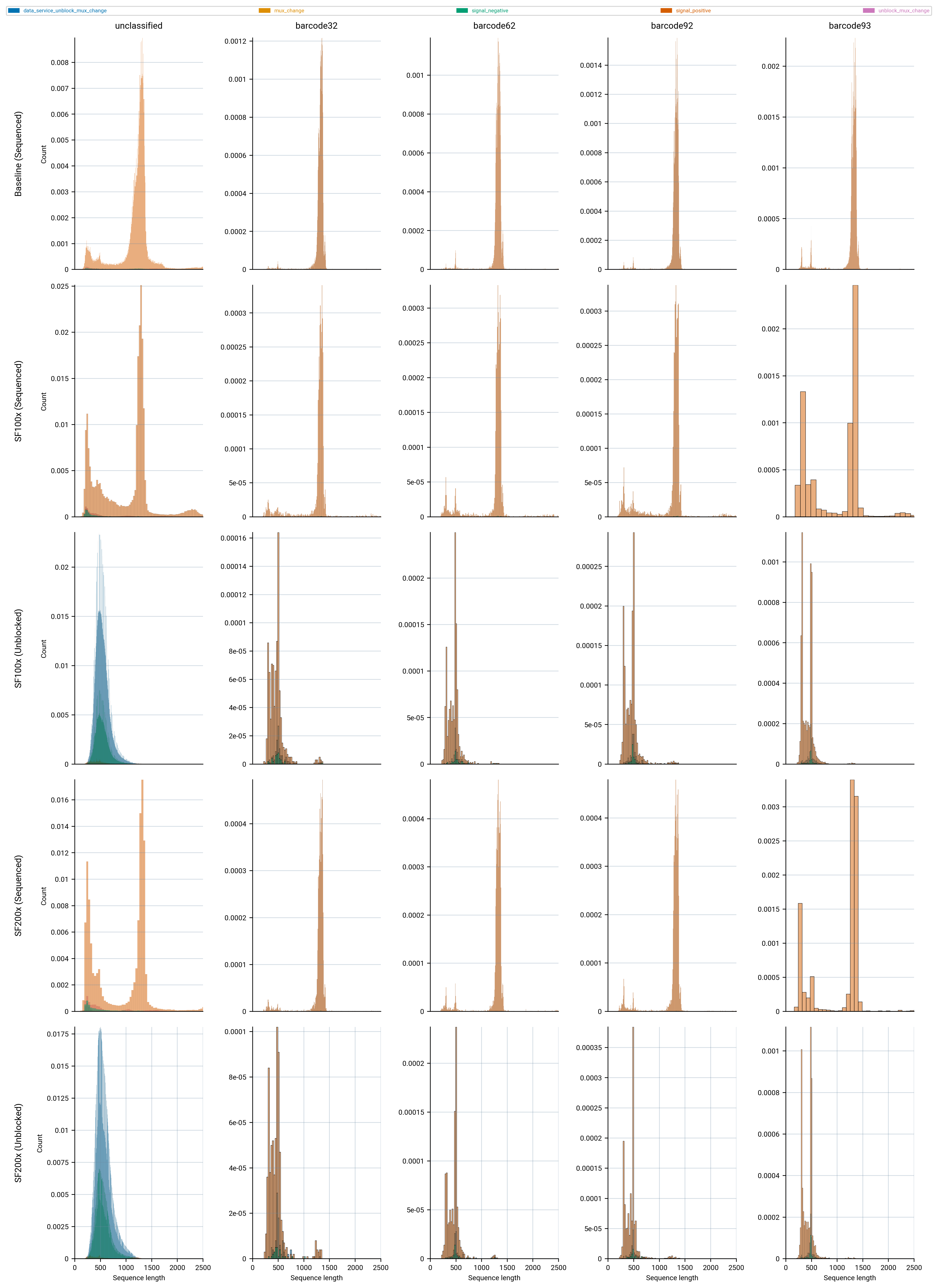

Supplement: Supplementary file 1 [file DataSheet1.ZIP › supplementary_data/Supplementary Info tex/Figures/sup_figure_10.png]

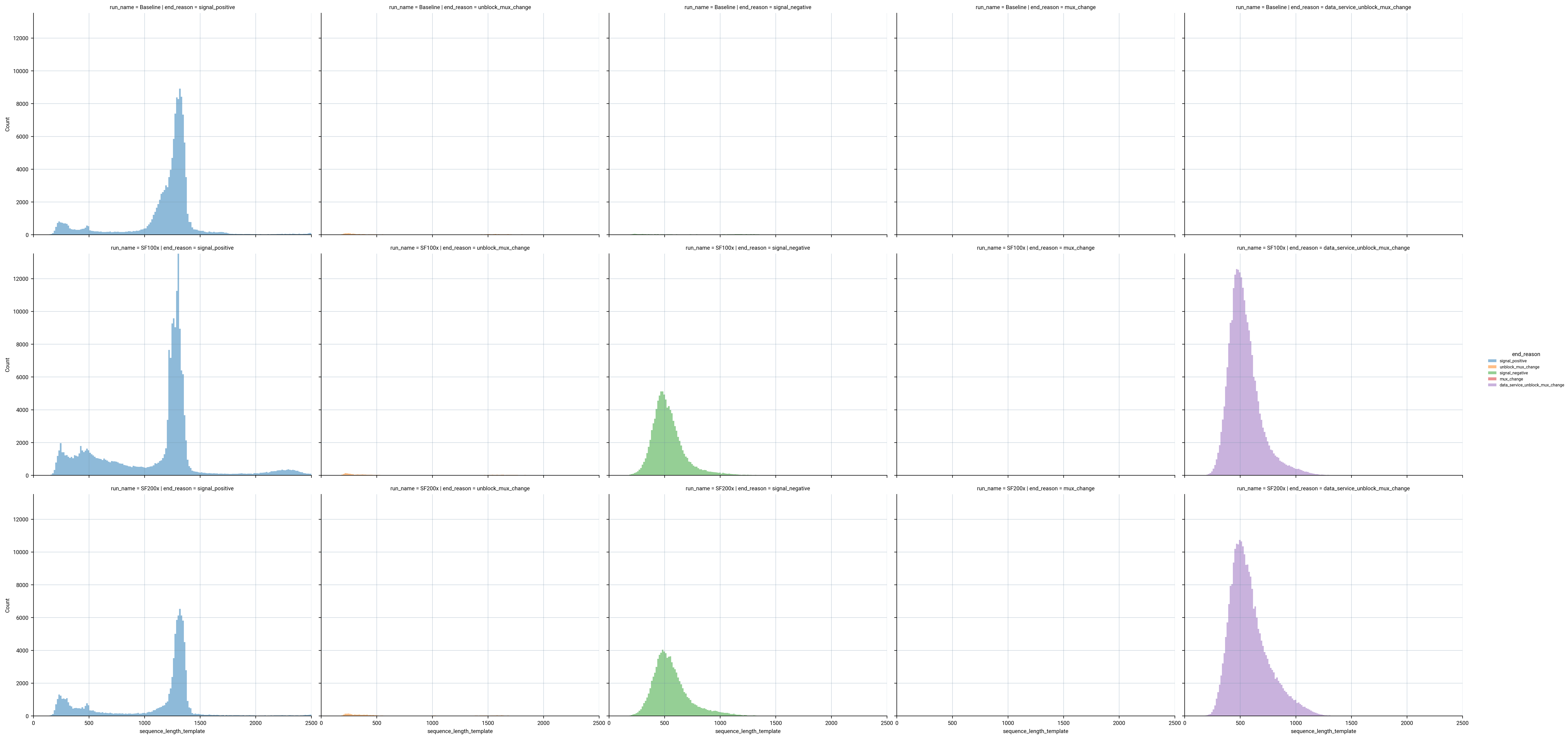

Supplement: Supplementary file 1 [file DataSheet1.ZIP › supplementary_data/Supplementary Info tex/Figures/sup_figure_9.png]

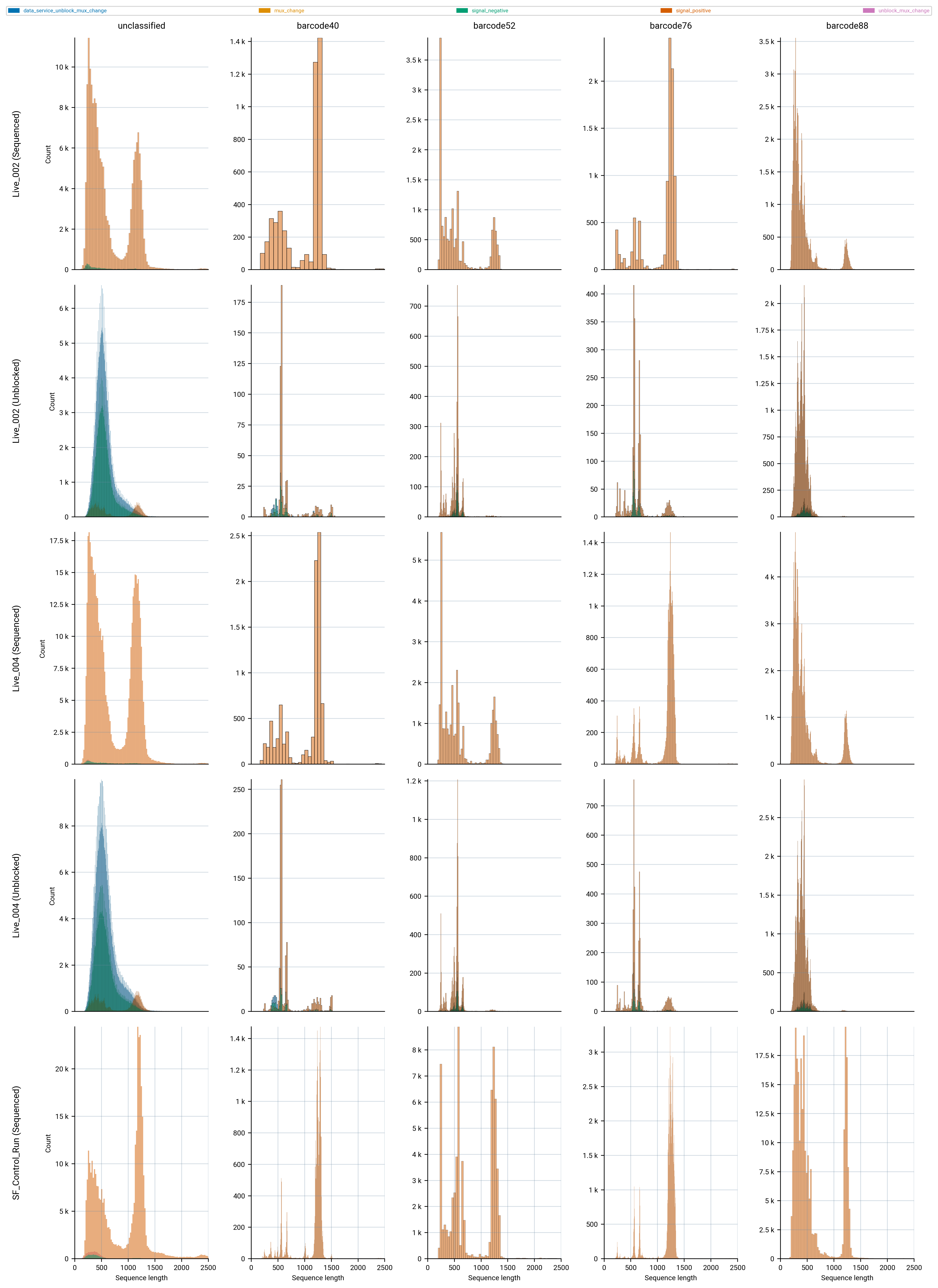

Supplement: Supplementary file 1 [file DataSheet1.ZIP › supplementary_data/Supplementary Info tex/Figures/sup_figure_5.png]

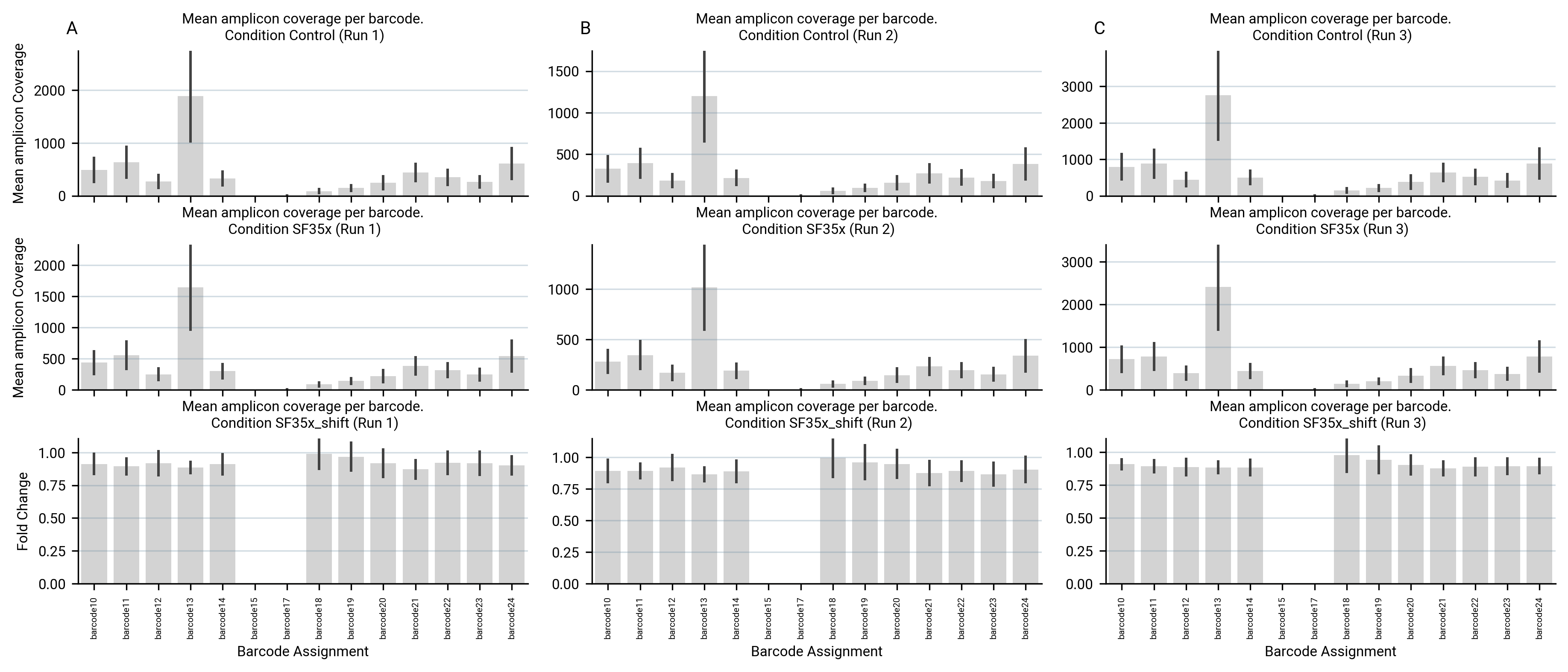

Supplement: Supplementary file 1 [file DataSheet1.ZIP › supplementary_data/Supplementary Info tex/Figures/figure_6_1.png]

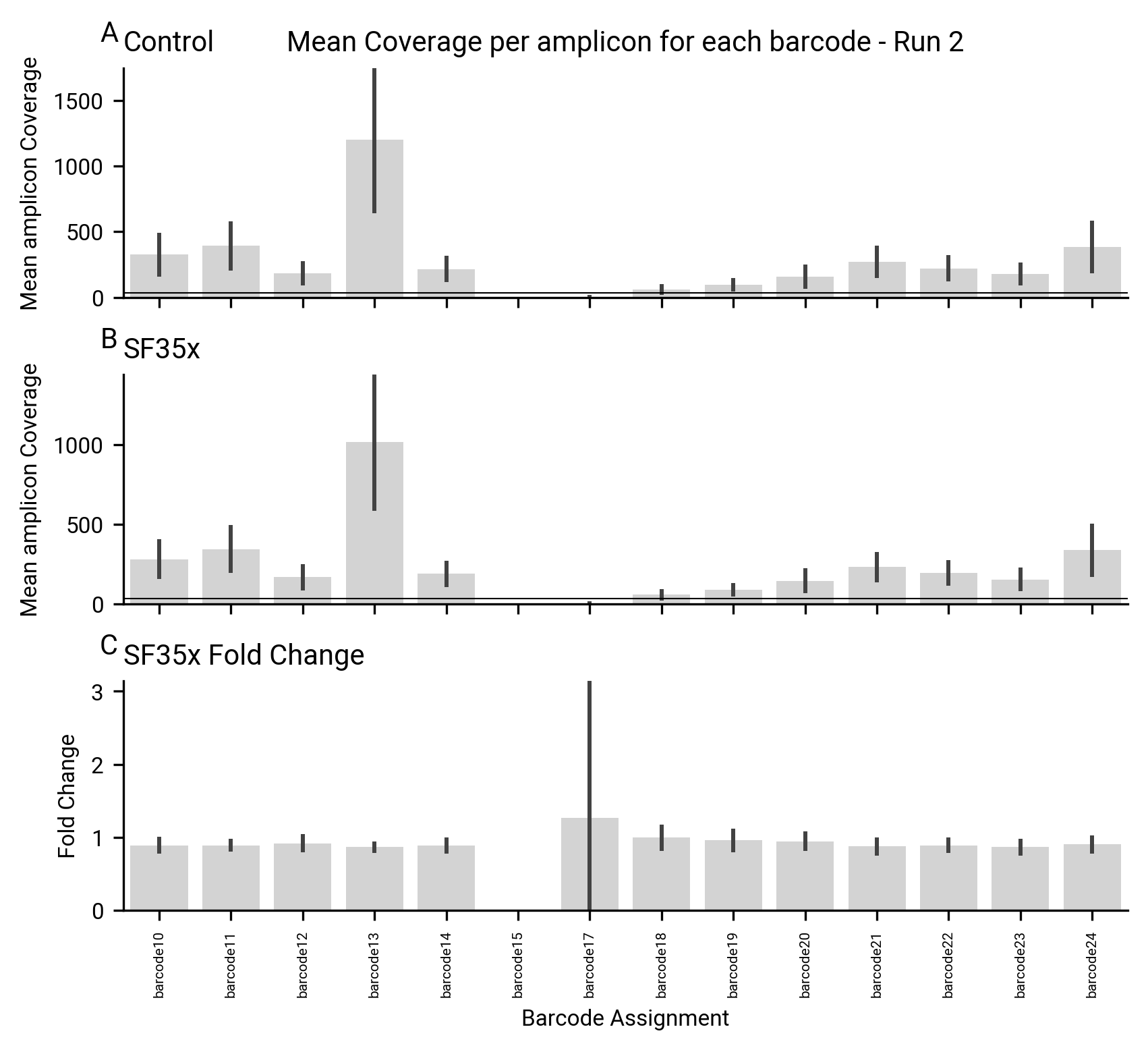

Supplement: Supplementary file 1 [file DataSheet1.ZIP › supplementary_data/Supplementary Info tex/Figures/figure_6_2.png]

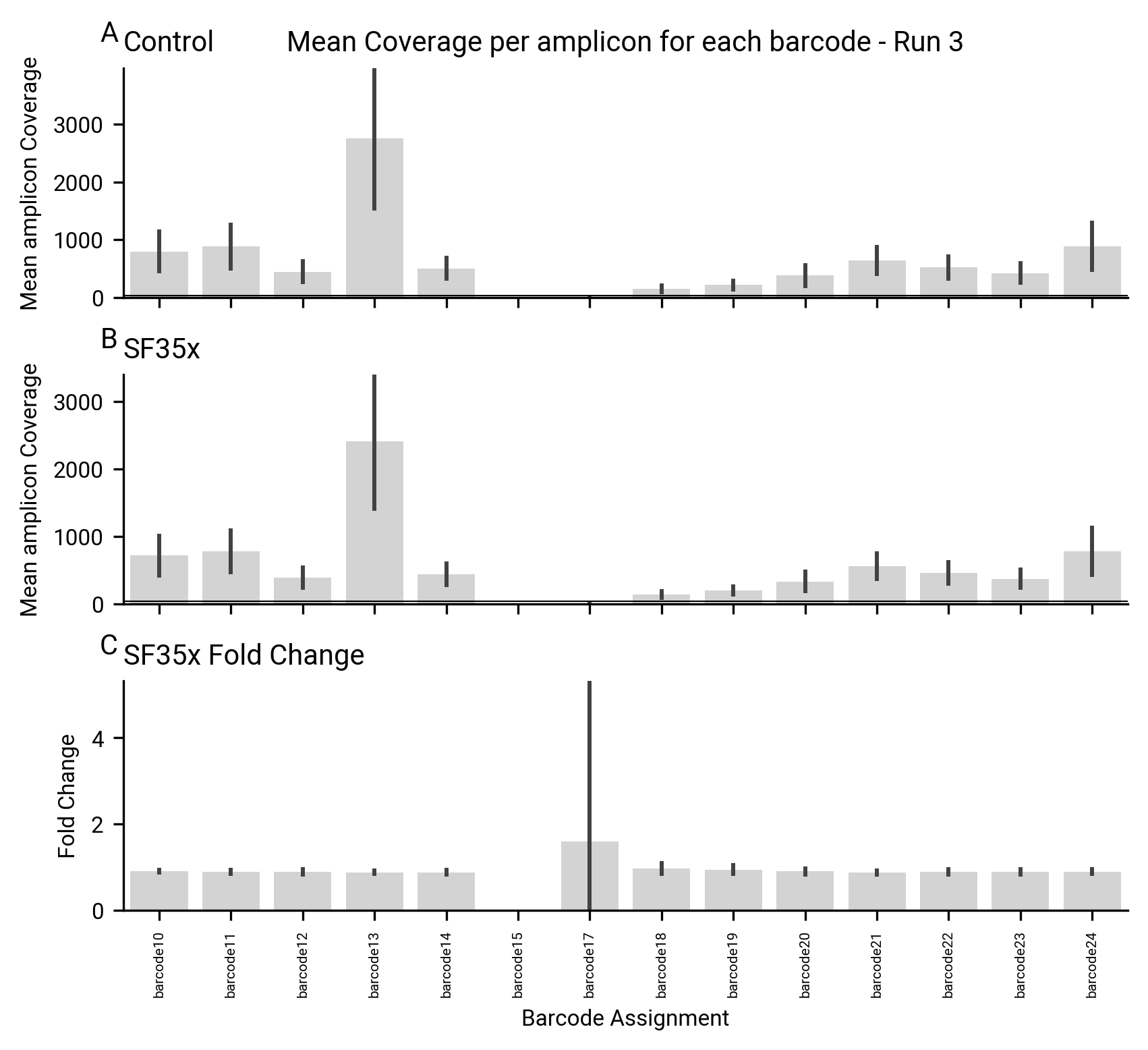

Supplement: Supplementary file 1 [file DataSheet1.ZIP › supplementary_data/Supplementary Info tex/Figures/figure_6_3.png]

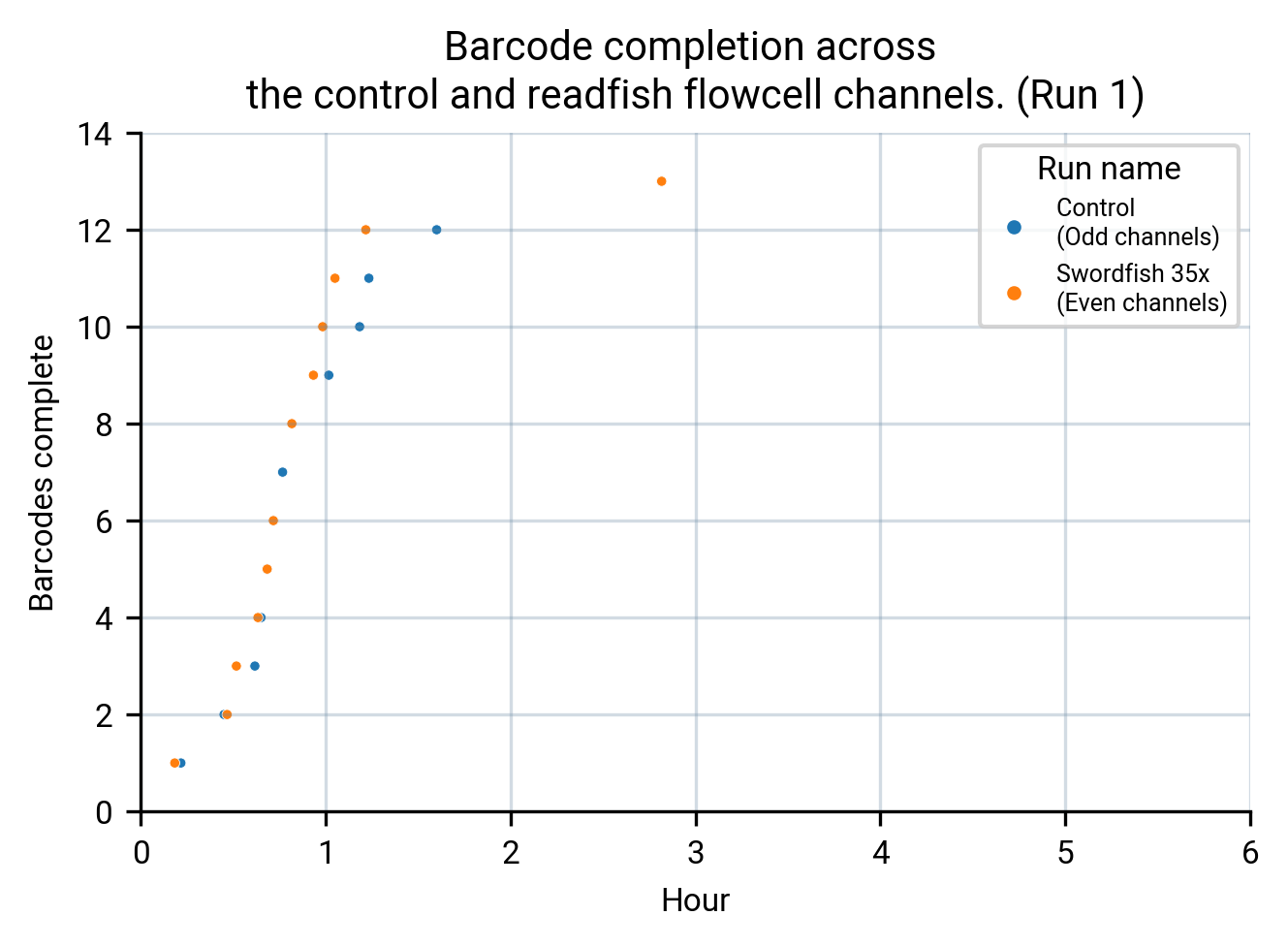

Supplement: Supplementary file 1 [file DataSheet1.ZIP › supplementary_data/Supplementary Info tex/Figures/figure_7_1.png]

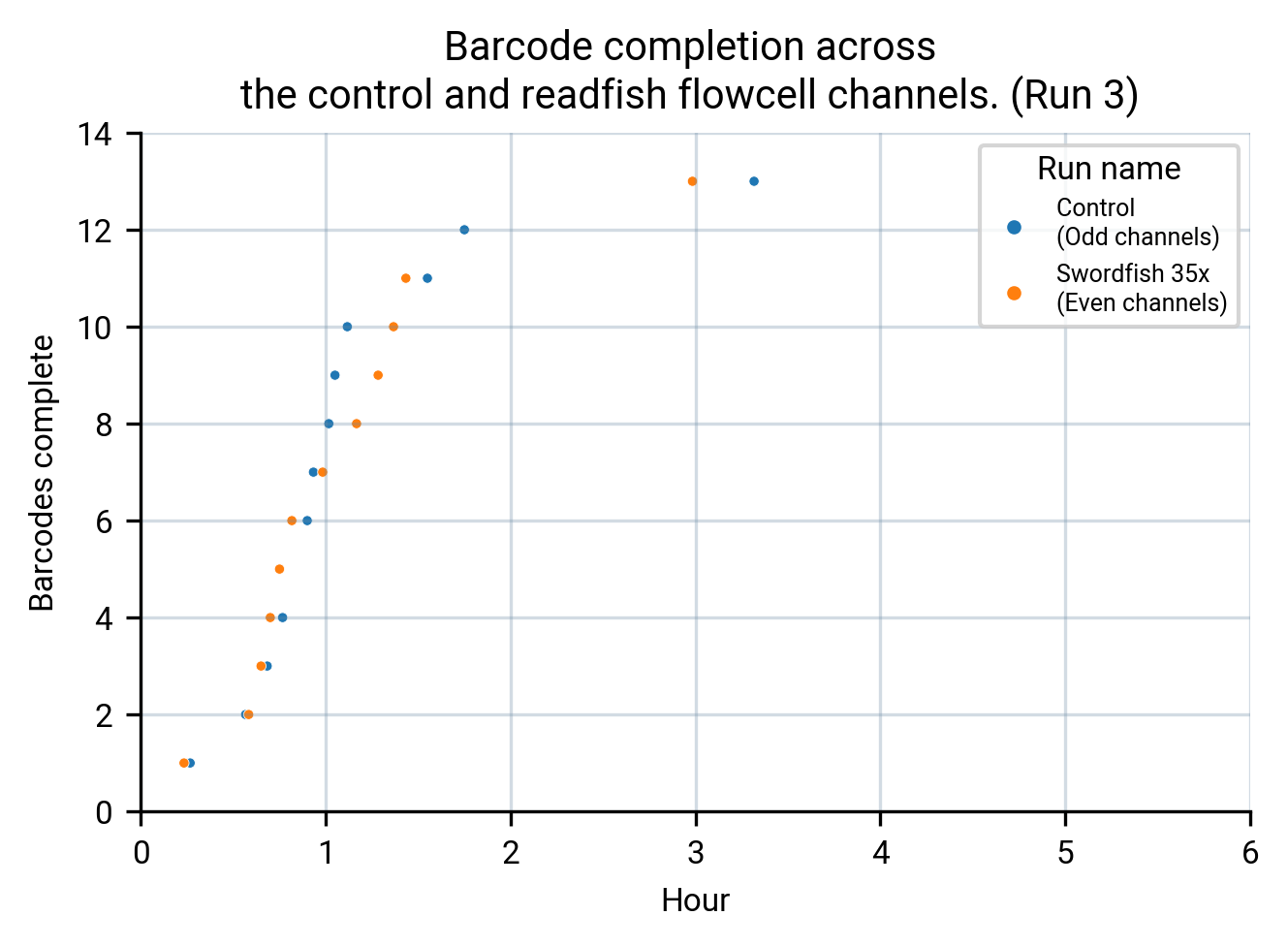

Supplement: Supplementary file 1 [file DataSheet1.ZIP › supplementary_data/Supplementary Info tex/Figures/figure_7_3.png]

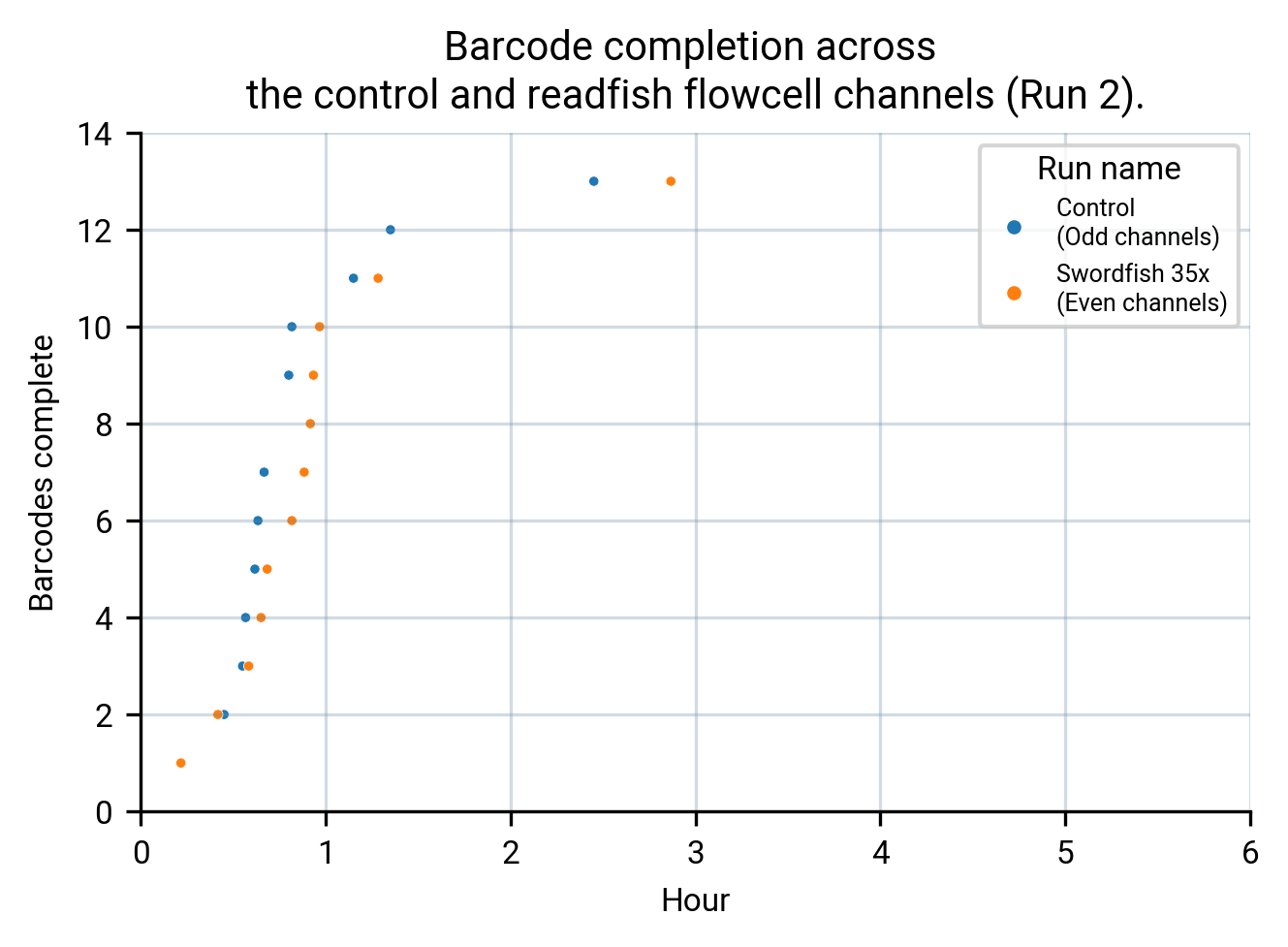

Supplement: Supplementary file 1 [file DataSheet1.ZIP › supplementary_data/Supplementary Info tex/Figures/figure_7_2.png]

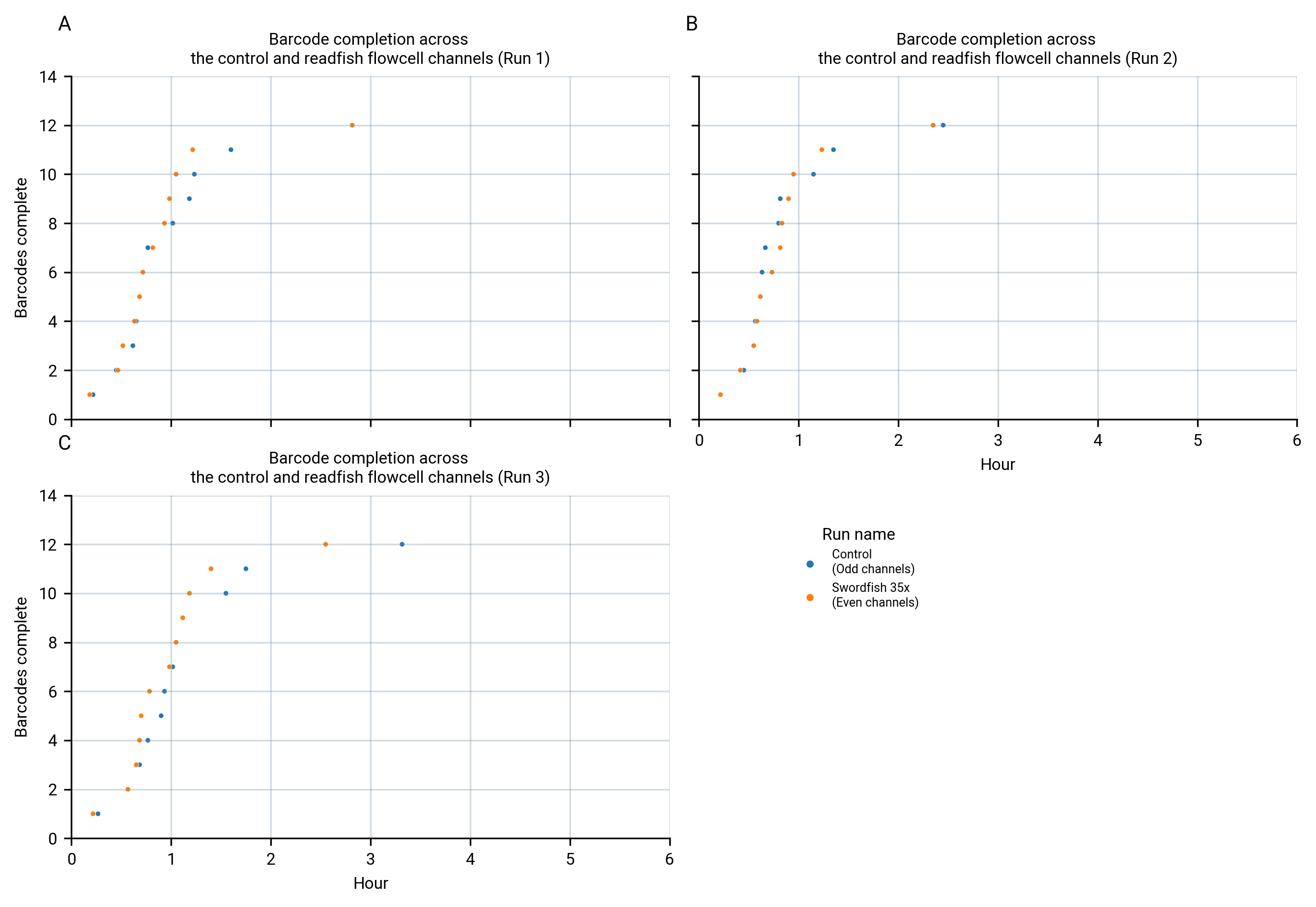

Supplement: Supplementary file 1 [file DataSheet1.ZIP › supplementary_data/Supplementary Info tex/Figures/sup_rapid_time.png]
