# Supplementary material for: A framework for real-time monitoring, analysis and adaptive sampling of viral amplicon nanopore sequencing
Source: Front Genet. 2023 Mar 27;14:1138582. doi: 10.3389/fgene.2023.1138582 (PMC10083257; doi:10.3389/fgene.2023.1138582)
Supplement: Supplementary file 1 [file DataSheet1.ZIP › supplementary_data/supplementary_file_1.tar.gz!/tmp/103_barcode25_artic_report.pdf]

Report for artic output of barcode25 generated 8th September 2021 14:18

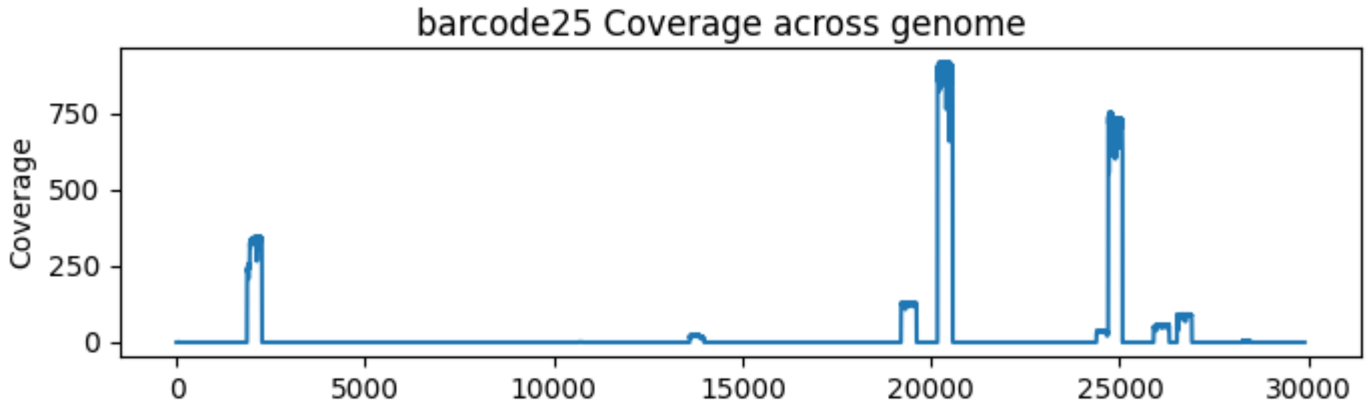

Artic variant summaries for barcode25

Variant of Concern Reports not available.

If a genome is complete and has a Variant of Concern lineage assigned to it, summary reports will be provided here based on current PHE Variant Definitions.
