# Supplementary material for: A framework for real-time monitoring, analysis and adaptive sampling of viral amplicon nanopore sequencing
Source: Front Genet. 2023 Mar 27;14:1138582. doi: 10.3389/fgene.2023.1138582 (PMC10083257; doi:10.3389/fgene.2023.1138582)
Supplement: Supplementary file 1 [file DataSheet1.ZIP › supplementary_data/supplementary_file_1.tar.gz!/tmp/103_barcode63_artic_report.pdf]

Report for artic output of barcode63 generated 8th September 2021 14:18

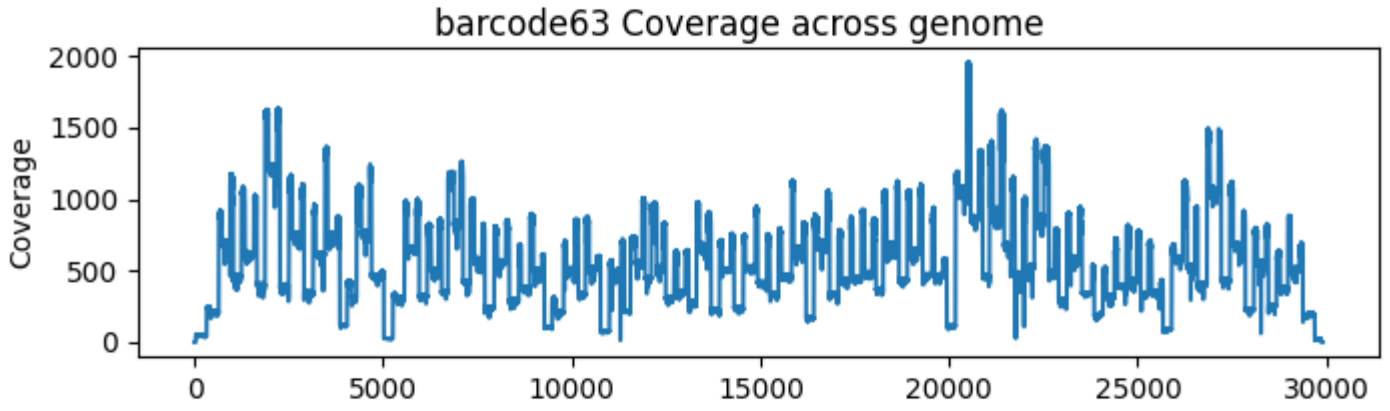

Artic variant summaries for barcode63

Variant of Concern Report for barcode63/ARTIC/medaka

If a genome is analysed, this will provide a report for the variant or variants found based on current PHE Variant Definitions. This analysis will report all possible VoCs and caution should be taken in interpretation of low coverage/quality genomes.

Reported VuI/VoCs:

sample_id	phe-label	unique-id	status	mutation-ref-calls	mutation-mixed-calls	mutation-calls	indel-ref-calls	indel-calls	no-calls	no-calls-deletion
barcode63/ARTIC/medaka	VOC-20DEC-01	denture-daughter	confirmed	0	0	13	0	2	0	0

Observed mutations:

	0	1	2	3	4	5	6	7	8	9	10
**sampleID**	barcode63/ARTIC/medaka	barcode63/ARTIC/medaka	barcode63/ARTIC/medaka	barcode63/ARTIC/medaka	barcode63/ARTIC/medaka	barcode63/ARTIC/medaka	barcode63/ARTIC/medaka	barcode63/ARTIC/medaka	barcode63/ARTIC/medaka	barcode63/ARTIC/medaka	barcode63/ARTIC/medaka
**type**	snp	snp	snp	snp	snp	snp	snp	snp	snp	snp	snp
**reference-base**	C	T	C	T	C	C	C	A	C	C	T
**variant-base**	T	C	T	C	T	T	T	G	A	T	C
**var-length**	1	1	1	1	1	1	1	1	1	1	1
**one-based-reference-position**	241	283	913	2698	3037	3267	3602	4964	5388	5986	6954
**iupac-variant-bases**	T	C	T	C	T	T	T	G	A	T	C

	11	12	13	14	15	16	17	18	19	20	21
**sampleID**	barcode63/ARTIC/medaka	barcode63/ARTIC/medaka	barcode63/ARTIC/medaka	barcode63/ARTIC/medaka	barcode63/ARTIC/medaka	barcode63/ARTIC/medaka	barcode63/ARTIC/medaka	barcode63/ARTIC/medaka	barcode63/ARTIC/medaka	barcode63/ARTIC/medaka	barcode6[illegible]/ARTIC/medaka
**type**	snp	del	snp	snp	snp	snp	snp	snp	del	del	snp
**reference-base**	C	GTCTGGTTTT	A	C	C	T	A	C	ATACATG	TTTA	A
**variant-base**	T	G	G	T	T	C	G	T	A	T	T
**var-length**	1	10	1	1	1	1	1	1	7	4	1
**one-based-reference-position**	7488	11287	12162	14408	15279	16176	17615	19386	21764	21990	23063
**iupac-variant-bases**	T	G	G	T	T	C	G	T	A	T	T

Sample_ID: barcode63/ARTIC/medaka
PANGO:B.1.1.7
nextstrain:N501Y.V1

Variant Status: confirmed

PHE-Label: VOC-20DEC-01
WHO Label: Alpha
Alternate Names: VOC202012/01, UK variant, Kent variant, VOC1,

Description:
This variant became widespread in the UK in the Winter of 2021 and is characterised by increased transmissibility.
Information Sources Source_1 Source_2

Variant Calls Detected:

Position	gene	protein	ref	variant	sample call	type	status
3267	ORF1ab	nsp3	C	T	T	SNP	detect
5388	ORF1ab	nsp3	C	A	A	SNP	detect
6954	ORF1ab	nsp3	T	C	C	SNP	detect
21764	S	surface glycoprotein	ATACATG	A	A	deletion	detect
21990	S	surface glycoprotein	TTTA	T	T	deletion	detect
23063	S	surface glycoprotein	A	T	T	SNP	detect
23271	S	surface glycoprotein	C	A	A	SNP	detect
23604	S	surface glycoprotein	C	A	A	SNP	detect
23709	S	surface glycoprotein	C	T	T	SNP	detect
24506	S	surface glycoprotein	T	G	G	SNP	detect
24914	S	surface glycoprotein	G	C	C	SNP	detect
27972	ORF8	ORF8 protein	C	T	T	SNP	detect
28048	ORF8	ORF8 protein	G	T	T	SNP	detect
28111	ORF8	ORF8 protein	A	G	G	SNP	detect
28280	N	nucleocapsid phosphoprotein	GAT	CTA	CTA	MNP	detect

One based position - notes on sites detected in this sample.

The observed calls were:

mutation_ref_calls: 0
indel_ref_calls: 0
mutation_calls: 13
mutation_mixed_calls: 0
indel_calls: 2
no_calls: 0
no_call_deletion: 0

Classification rules:

confirmed

mutations_required: 13

indels_required: 0

allowed_wildtype: 0

probable

mutations_required: 5

indels_required: 0

allowed_wildtype: 0

low_qc

mutations_required: 0

indels_required: 0

allowed_wildtype: 0

Acknowledgements:
*Curators:*Natalie Groves Ulf Schaefer Nick Loman

The complete list of variants of concern scanned included:
VUI-21FEB-04 VOC-20DEC-02 VUI-21FEB-01 VUI-21MAR-01 VUI-21MAY-01 VOC-20DEC-01 VOC-21APR-02 VUI-21FEB-03 VUI-21APR-01 E484K VUI-21JUL-01 VUI-21JAN-01 VUI-21MAR-02 VUI-21MAY-02 VUI-21JUN-01 VOC-21JAN-02 VUI-21APR-03 VOC-21FEB-02
