# Supplementary material for: A framework for real-time monitoring, analysis and adaptive sampling of viral amplicon nanopore sequencing
Source: Front Genet. 2023 Mar 27;14:1138582. doi: 10.3389/fgene.2023.1138582 (PMC10083257; doi:10.3389/fgene.2023.1138582)
Supplement: Supplementary file 1 [file DataSheet1.ZIP › supplementary_data/supplementary_file_1.tar.gz!/tmp/103_artic_report.pdf]

Report for artic output of FAQ01023_CV197_25_M1 generated 8th September 2021 14:18

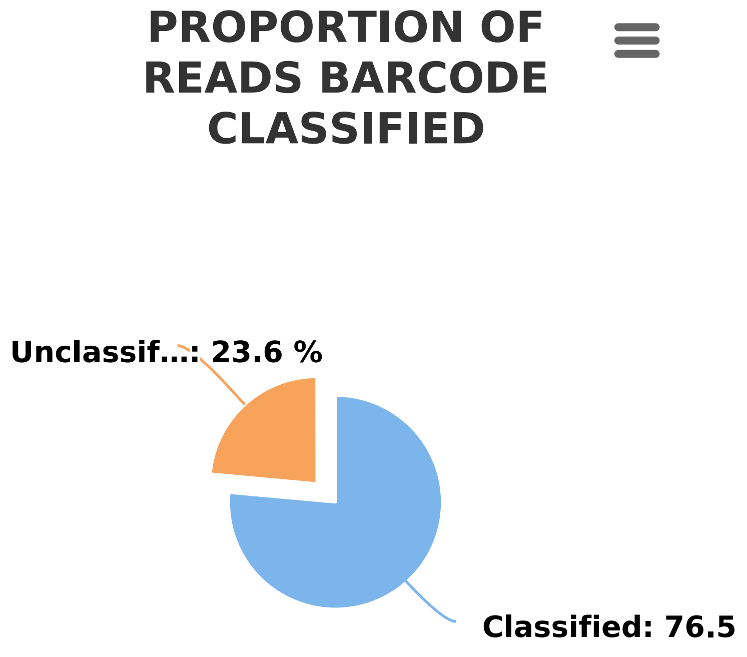

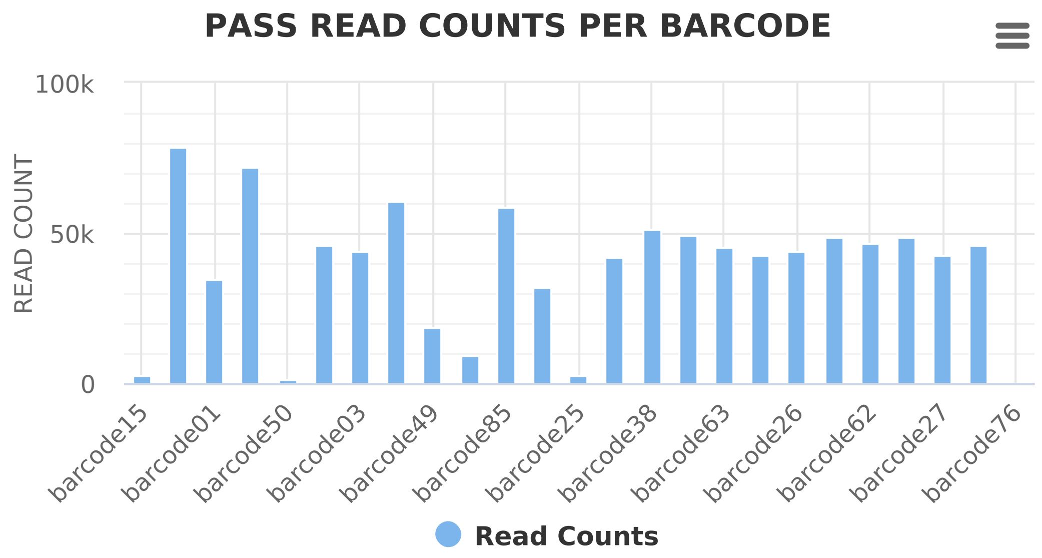

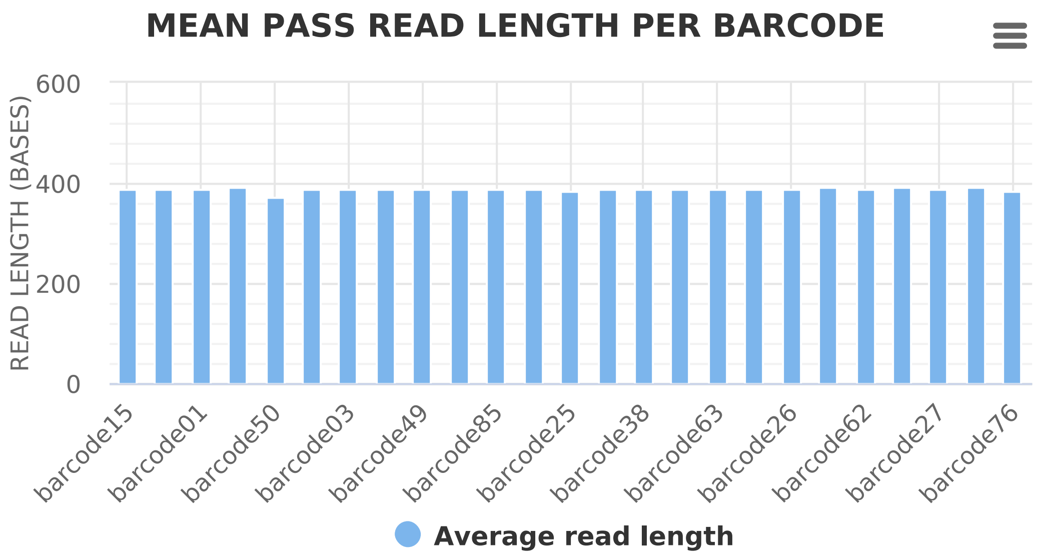

Barcode Name	Chromosome	Read Len	Read Count	Yield	Coverage	# Success Amplicons	# Partial Amplicons	# Failed Amplicons	Lineage	VoC found
barcode01	MN908947.3	388	34,489	13,393,773	438.88	76	4	19	Currently unknown	Not Tested
barcode02	MN908947.3	388	49,127	19,051,766	624.2	99	0	0	B.1.1.7	0 VOC-20DEC-01 Name: phe-label, dtype: object
barcode03	MN908947.3	389	44,022	17,106,691	560.4	94	5	0	Currently unknown	Not Tested
barcode13	MN908947.3	388	32,321	12,537,233	410.86	88	9	2	Currently unknown	Not Tested
barcode14	MN908947.3	391	45,820	17,913,802	587.57	99	0	0	B.1.1.7	0 VOC-20DEC-01 Name: phe-label, dtype: object
barcode15	MN908947.3	388	2,415	936,286	30.72	17	5	77	Currently unknown	Not Tested
barcode25	MN908947.3	386	2,348	906,274	29.77	8	2	89	Currently unknown	Not Tested
barcode26	MN908947.3	390	44,063	17,173,790	563.16	99	0	0	B.1.1.7	0 VOC-20DEC-01 Name: phe-label, dtype: object

Barcode Name	Chromosome	Read Len	Read Count	Yield	Coverage	# Success Amplicons	# Partial Amplicons	# Failed Amplicons	Lineage	VoC found
barcode27	MN908947.3	387	42,641	16,520,274	541.29	96	3	0	Currently unknown	Not Tested
barcode37	MN908947.3	387	46,152	17,881,875	585.97	91	5	3	Currently unknown	Not Tested
barcode38	MN908947.3	388	51,030	19,810,123	649.61	98	0	1	B.1.1.7	0 VOC-20DEC-01 Name: phe-label, dtype: object
barcode39	MN908947.3	388	42,319	16,437,763	538.77	95	4	0	B.1.1.7	0 VOC-20DEC-01 Name: phe-label, dtype: object
barcode49	MN908947.3	387	18,946	7,325,827	240.15	63	7	29	Currently unknown	Not Tested
barcode50	MN908947.3	374	1,506	563,440	18.46	16	3	80	Currently unknown	Not Tested
barcode51	MN908947.3	391	72,218	28,269,815	927.41	99	0	0	B.1.1.7	0 VOC-20DEC-01 Name: phe-label, dtype: object
barcode61	MN908947.3	388	9,602	3,724,731	122.27	34	4	61	Currently unknown	Not Tested
barcode62	MN908947.3	390	46,897	18,282,670	599.62	99	0	0	B.1.1.7	0 VOC-20DEC-01 Name: phe-label, dtype: object
barcode63	MN908947.3	390	45,320	17,654,226	579.03	99	0	0	B.1.1.7	0 VOC-20DEC-01 Name: phe-label, dtype: object
barcode73	MN908947.3	391	48,380	18,893,415	619.56	99	0	0	B.1.1.7	0 VOC-20DEC-01 1 E484K Name: phe-label, dtype: object
barcode74	MN908947.3	390	42,751	16,659,804	546.41	99	0	0	B.1.1.7	0 VOC-20DEC-01 Name: phe-label, dtype: object
barcode75	MN908947.3	389	60,771	23,647,077	774.72	97	2	0	B.1.1.7	0 VOC-20DEC-01 Name: phe-label, dtype: object
barcode76	MN908947.3	384	1	384	0.01	0	1	98	Currently unknown	Not Tested
barcode85	MN908947.3	389	58,861	22,924,435	751.68	99	0	0	B.1.1.7	0 VOC-20DEC-01 Name: phe-label, dtype: object
barcode86	MN908947.3	392	48,633	19,063,992	625.28	99	0	0	B.1.1.7	0 VOC-20DEC-01 Name: phe-label, dtype: object
barcode87	MN908947.3	389	78,605	30,562,375	1,002.43	99	0	0	B.1.1.7	0 VOC-20DEC-01 Name: phe-label, dtype: object
unclassified	MN908947.3	369	298,621	110,329,619	3,595.19	99	0	0	Currently unknown	Not Tested

Artic generated SNIPIT plot and tree for FAQ01023_CV197_25_M1

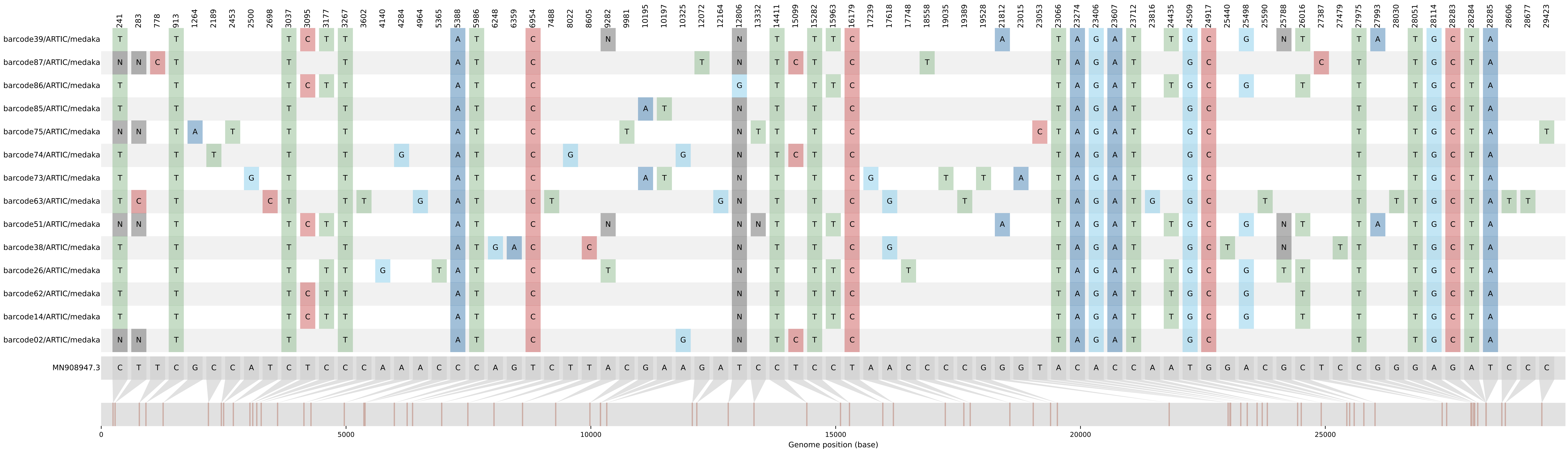

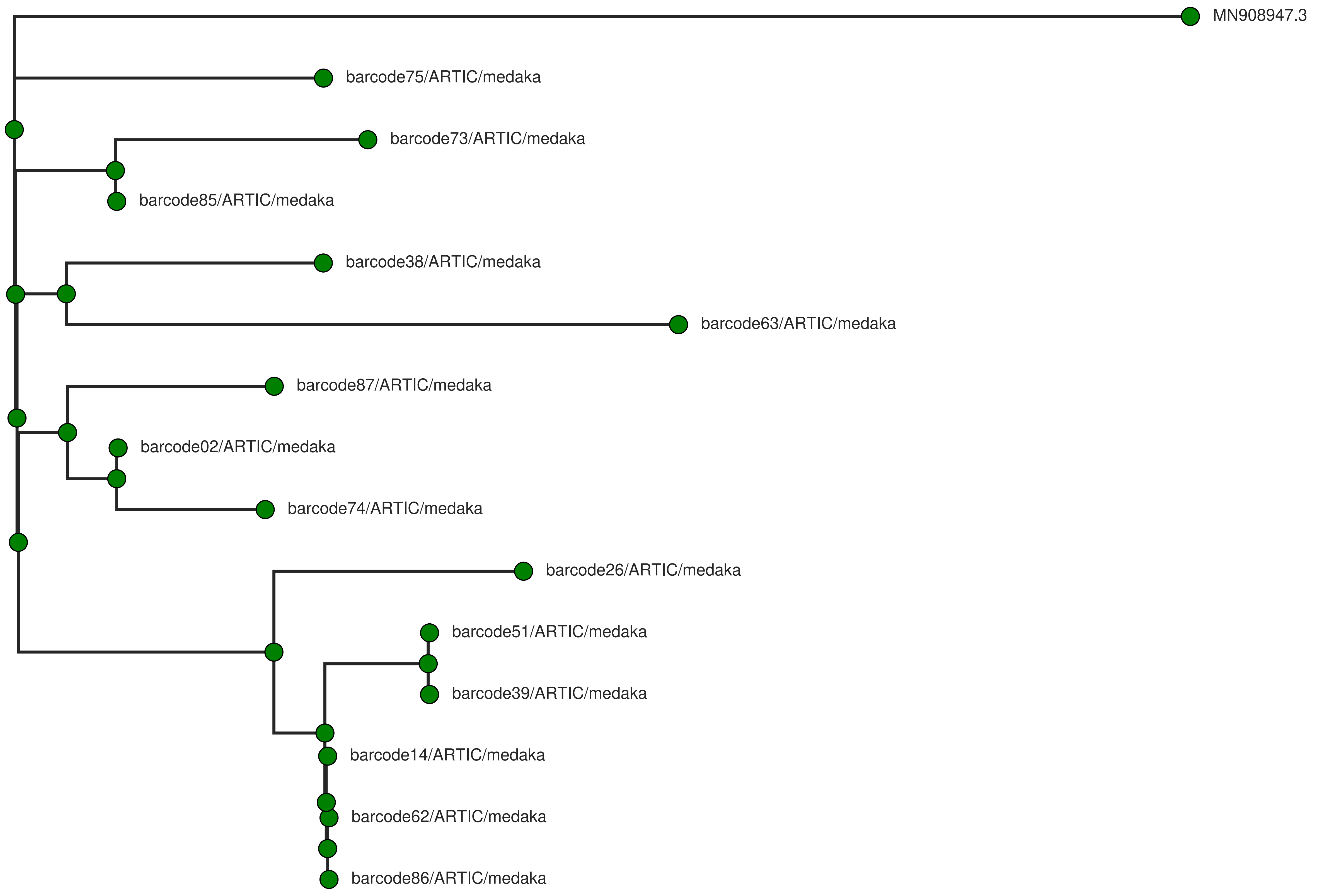
MN908947.3
barcode75/ARTIC/medaka
barcode73/ARTIC/medaka
barcode85/ARTIC/medaka
barcode38/ARTIC/medaka
barcode63/ARTIC/medaka
barcode87/ARTIC/medaka
barcode02/ARTIC/medaka
barcode74/ARTIC/medaka
barcode26/ARTIC/medaka
barcode51/ARTIC/medaka
barcode39/ARTIC/medaka
barcode14/ARTIC/medaka
barcode62/ARTIC/medaka
barcode86/ARTIC/medaka
